# Oxygenated Solanapyrone Analogs From *Nigrospora* sp. IQ‐064, a Mangrove Associated Fungus

**DOI:** 10.1002/cbdv.202501912

**Published:** 2025-08-25

**Authors:** Carlos A. Fajardo‐Hernández, Ángel Sahid Aguilar‐Colorado, Leslie Maribel Corona‐Cabello, Ingrid Y. Martínez‐Aldino, José Rivera‐Chávez

**Affiliations:** ^1^ Instituto de Química, Departamento de Productos Naturales Universidad Nacional Autónoma de México Mexico City Mexico

**Keywords:** metabolomics, natural products, *Nigrospora*, polyketides, solanapyrone

## Abstract

Two new oxygenated solanapyrone analogues, nigrosporapyrone E (**1**) and nigrosporapyrone F (**2**), were isolated from *Nigrospora* sp. strain IQ‐064, a fungus associated with the bark of black mangrove (*Avicennia germinans* L.), along with seven known compounds. A targeted metabolomic strategy, on the basis of molecular networking and chemotaxonomic criteria, facilitated the isolation of these compounds. Their structures were characterized using HRESIMS and NMR spectroscopic analysis, whereas their relative configurations were established through NOE correlations. The absolute configuration of the new natural products was determined by comparison of experimental and calculated electronic circular dichroism (ECD) spectra, showing consistency with the recently reported natural product sphasolanapyrone A. Considering previous bioprospecting studies of this fungal strain, the isolated compounds were evaluated for their ability to inhibit human protein tyrosine phosphatase 1B (*h*PTP1B_1–400_), a validated target for developing anti‐diabetic drugs, as well as for their inhibitory activity against the pathogen *Acinetobacter baumannii* clinical isolate A564. However, no significant activity was observed in either bioassay. In addition, their ADME properties and probable bioactivity spectrum, predicted through SwissADME and PASS analysis, respectively, were analyzed to investigate alternative molecular targets within this compound family.

## Graphical abstract

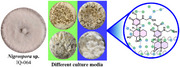

## 1 Introduction

*Nigrospora* strains are commonly found as plant pathogens, endophytes, or saprobes and are recognized as promising sources of novel molecules [1]. Metabolites from *Nigrospora* have been isolated from terrestrial and marine environments in roughly equal proportions. Most of these metabolites are polyketides and exhibit diverse biological activities, including cytotoxic, antifungal, antibacterial, antiviral, antioxidant, anti‐inflammatory, antimalarial, phytotoxic, and enzyme inhibitory properties [2]. Notably, the solanapyrone family—also referred to as nigrosporapyrones or sphasolanapyrones, depending on the producing organism—stands out due to its structural complexity. These compounds feature a 3,4‐dehydrodecalin moiety formed via an intramolecular [4 + 2] cycloaddition reaction catalyzed by the enzyme solanapyrone synthase, a Diels–Alderase that strongly favors the formation of the *exo*‐adduct [3, 4].

Previously, Martínez‐Aldino et al. described the prioritization of several mangrove‐associated fungal strains, using a multi‐informative approach combining non‐targeted metabolomics, biological activity, and taxonomical data. This methodology selected the extract of *Nigrospora* sp. strain IQ‐064, isolated from the bark of black mangrove (*Avicennia germinans*) from Tamiahua, El Ídolo Lagoon in Mexico, based on its metabolic profile and the probability of isolating interesting chemical entities considering a chemotaxonomic criteria [5]. In addition, the fungus exhibited promising activity against long‐chain protein tyrosine phosphatase 1B (*h*PTP1B_1–400_), a validated target for the development of anti‐diabetic drugs, as well as inhibitory effects on the growth of multidrug‐resistant *Acinetobacter baumannii* clinical isolate A564, a pathogen responsible for infections that are difficult to treat [5].

To explore the chemical diversity of this species, a comprehensive study was conducted. This involved conducting a metabolomic analysis of the organic extracts obtained from fungi growth on two substrates (rice and Cheerios cereal), followed by a conventional chemical investigation. This approach led to the discovery of two new oxygenated solanapyrones (**1** and **2**), along with seven previously known compounds (**3**–**9**). Their structures, including absolute configuration, were elucidated using a set of spectrometric (HRESIMS), spectroscopic (NMR), and chiroptical methods (electronic circular dichroism [ECD]). All isolates were tested for their inhibitory potential towards *h*PTP1B_1–400_ enzyme. Moreover, their absorption, distribution, metabolism, excretion, and toxicity (ADME‐Tox) properties were predicted using in silico tools, and a potential bioactivity spectrum was explored through PASS (prediction of activity spectra for substances) analysis.

## 2 Results and Discussion

**FIGURE 1:**
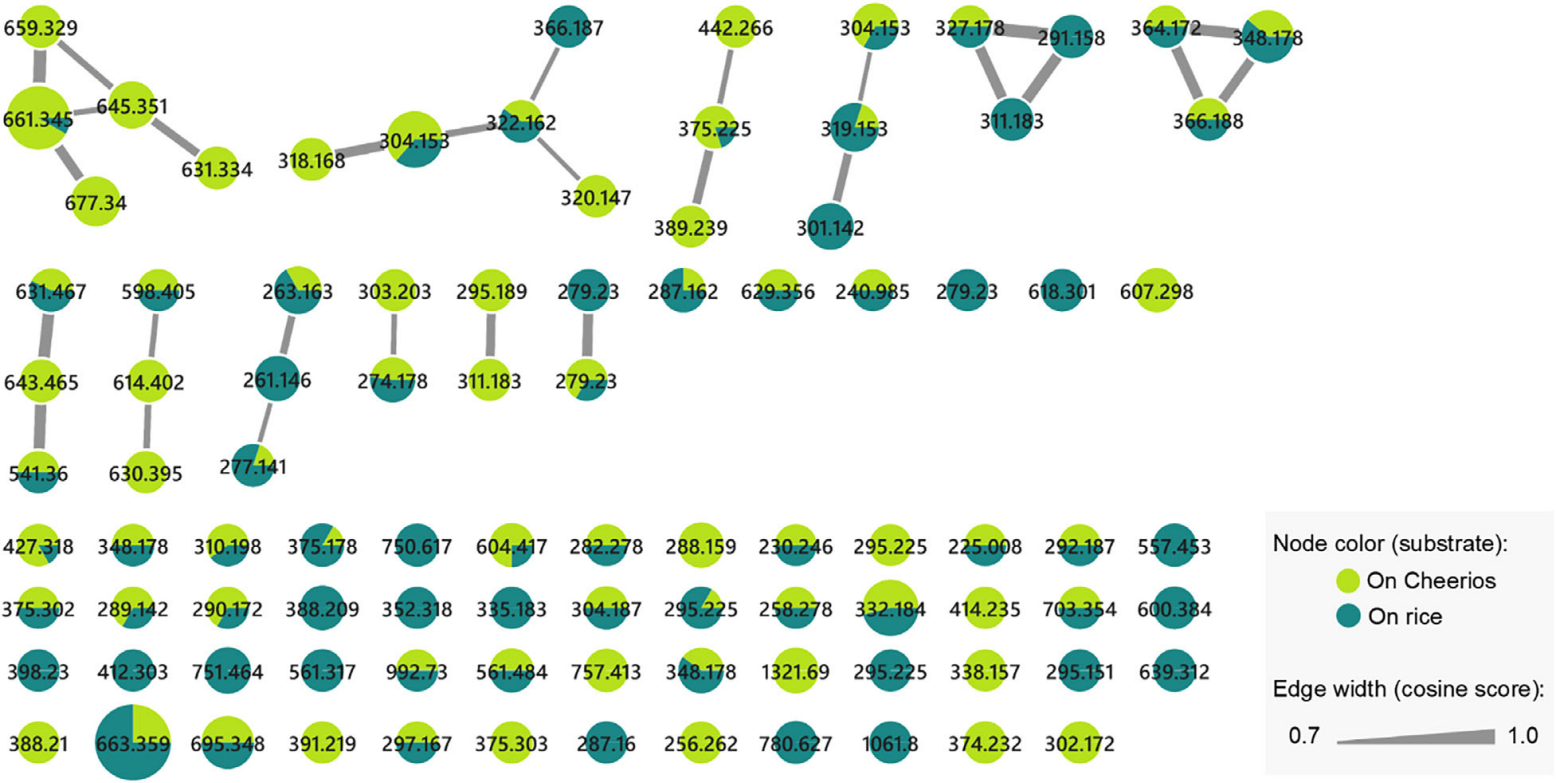
Molecular network of metabolites produced by *Nigrospora* sp. IQ‐064 cultivated on two different substrates generated using the GNPS platform. Each node represents a parent ion (feature), and its corresponding *m/*z value is shown within the node. Node size is proportional to the total ion intensity, calculated from the number of MS/MS spectra used to build each node. Edges (connections between nodes) indicate structural similarity on the basis of MS/MS fragmentation patterns; thicker edges represent higher cosine similarity values (ranging from 0.7 to 1.0). Nodes are color‐coded according to the substrate in which they were predominantly detected.

To expand the metabolome produced by the fungus of interest, it was cultivated on Cheerios and rice substrates, and its chemical composition was analyzed by molecular networking (Figure 1), where each consensus node represents a putative compound with a specific parent ion. A total of 94 nodes were detected; 57 remained as singletons, whereas the remaining (37) were grouped into molecular families. Almost half of the nodes (43) were detected in both growth conditions. The rest were distributed in similar proportions for each media (27 and 24, detected on Cheerios and rice, respectively). Notably, the Cheerios‐derived extract produced more prominent compound families, as it was represented in greater number of molecular clusters. This observation was further supported by HPLC‐PDA analysis of both extracts, which revealed a greater number of signals of interest in the extract obtained from Cheerios medium.

**SCHEME 1:**
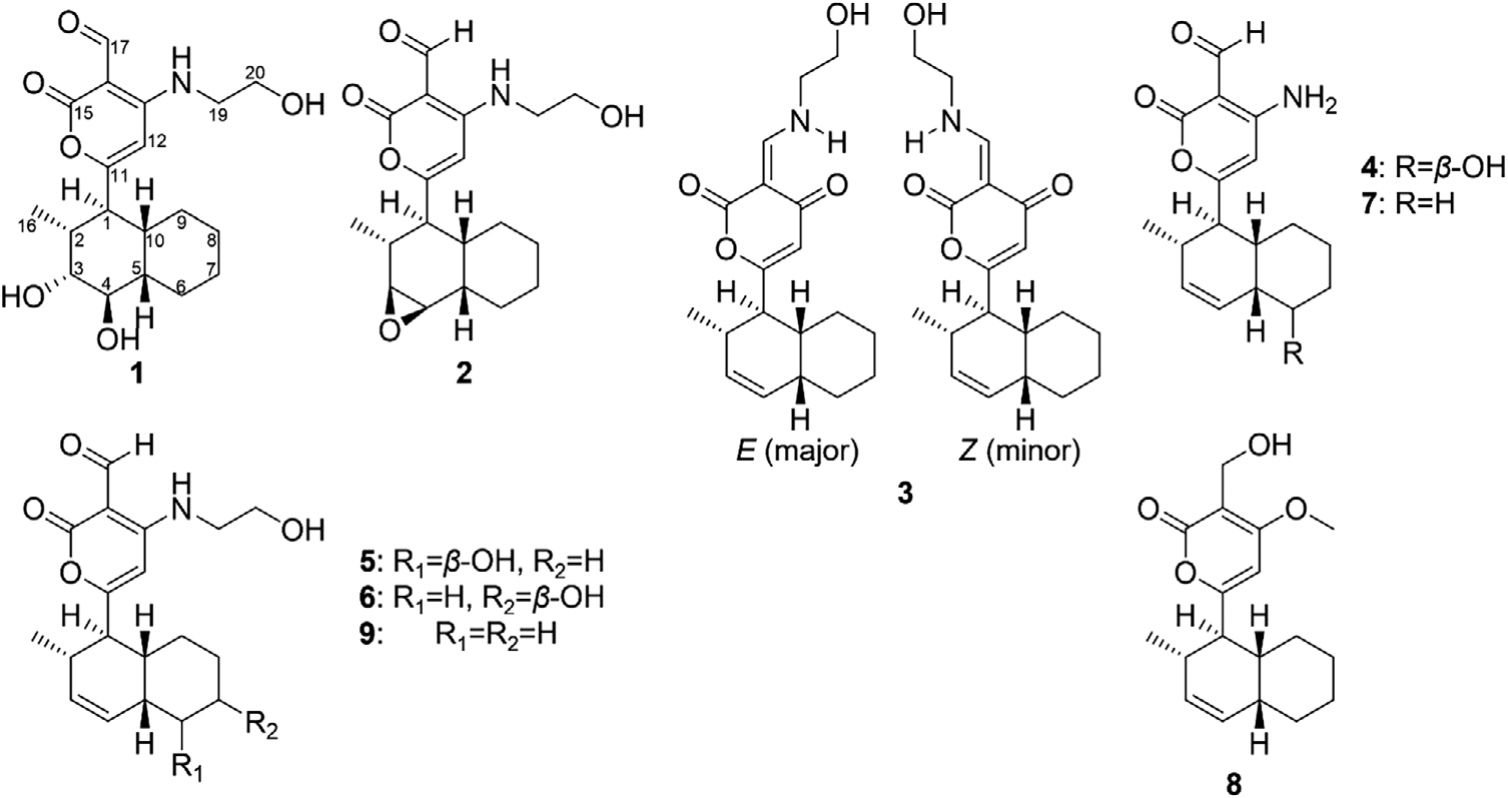
Isolated compounds from *Nigrospora* sp. IQ‐064.

As the Global Natural Product Social (GNPS) database did not yield significant matches with the dataset, a manual dereplication of the observed *m*/*z* values in the molecular network was performed. This process considered three conserved UV maxima observed at approximately 220–230, 270–280, and 315–330 nm by HPLC‐PDA analysis. Several databases were consulted, including the Dictionary of Natural Products (https://dnp.chemnetbase.com/), LOTUS (https://lotus.naturalproducts.net/) [6], and the Natural Products Atlas (https://www.npatlas.org/) [7], under the assumption that the most probable scaffold corresponded to solanapyrone analogs, on the basis of both UV data and chemotaxonomic criteria. Given the potential of the fungus to produce new compounds and the presence of certain *m*/*z* features lacking clear matches in any of the consulted databases, combined with the contribution of the Cheerios substrate to generate structural diversity, this medium was selected and scaled‐up for detailed chemical investigation. After successive partitioning procedures, nine compounds were yielded, two of which were identified as new natural products (**1** and **2**; Scheme 1). The structural elucidation of these novel compounds is described below.

Nigrosporapyrone E (**1**) was isolated as a white solid from acetone. Its molecular formula was determined to be C_19_H_27_NO_6_, based on *m/z* 364.1758 [M‐H]^−^ (calcd for C_19_H_26_NO_6_, 364.1755, *Δ* + 0.9 ppm), indicating seven degrees of unsaturation. The ^13^C‐NMR spectrum of **1** (Table 1) revealed the presence of two carbonyl groups, one for an aldehyde at *δ*_C_ 190.6 ppm (C‐17) and one for an ester at *δ*_C_ 164.2 ppm (C‐15), along with two downfield signals corresponding to *sp* ^2^ carbons at *δ*_C_ 161.3 ppm (C‐13) and 171.7 ppm (C‐11), and two upfield signals assignable to *sp* ^2^ carbons at *δ*_C_ 95.2 ppm (C‐14) and 98.0 ppm (C‐12). The signal at *δ*_C_ 171.7 was barely detected by its HMBC correlation with the olefinic proton at *δ*_H_ 6.52 (s, H‐12). Additionally, a nitrogen‐bonded methylene was observed at *δ*_C_ 46.1 ppm (C‐19), along with an aliphatic oxygen‐bonded methylene at *δ*_C_ 61.2 ppm (C‐20).

Analysis of the ^1^H‐NMR spectrum (Table 1) revealed signals corresponding to an amine (*δ*_H_ 10.76), an aldehyde (*δ*_H_ 9.88), an olefinic proton (*δ*_H_ 6.52), two oxygenated methines (*δ*_H_ 4.17 and *δ*_H_ 3.90), two heteroatom‐bonded methylenes (*δ*_H_ 3.80 and *δ*_H_ 3.66), one methyl group (*δ*_H_ 0.92), and several aliphatic *sp* ^3^ CH and CH_2_ signals. On the basis of this spectroscopic information, a decalin moiety was proposed, as in solanapyrones. Notably, in the ^1^H‐NMR spectrum of **1**, the absence of the two vinylic carbon signals typically present in solanapyrone analogues was evident. Instead, two oxygen‐based methines were detected at *δ*_H_/*δ*_C_ 3.90/75.1 ppm (C‐3) and 4.17/65.7 ppm (C‐4).

**TABLE 1:** ^1^H (700 MHz) and ^13^C (150 MHz) NMR data of compounds **1** (acetone‐*d*_6_) and **2** (CDCl_3_).

	1		2	
No.	*δ*_C_ type	*δ*_H_, mult (*J* in Hz)	*δ*_C_ type	*δ*_H_, mult (*J* in Hz)
1	43.2, CH	2.88, dd (11.6, 11.6)	45.6, CH	2.20, d‐broad (9.6)^a^
2	33.7, CH	2.44, m	33.1, CH	2.30, p‐broad (7.3)
3	75.1, CH	3.90, t‐broad (3.5)	56.2, CH	2.90, d (3.9)
4	65.7, CH	4.17, dd‐broad (1.8, 1.8)	57.7, CH	3.00, dd (3.9, 2.0)
5	44.8, CH	2.09, m^a^	35.8, CH	2.25, dd (12.7, 2.0)
6	28.5, CH_2_	2.14, dtd (13.3, 13.3, 3.8)	25.8, CH_2_	1.81, d‐broad (11.9)
		1.57, dd‐broad (13.3, 3.4)		1.31, m^a^
7	27.8, CH_2_	1.72, d‐broad (12.9)	27.9, CH_2_	1.43, m^a^
		1.20, qt (12.9, 4.0)		1.31, m^a^
8	21.4, CH_2_	1.44, qt (13.1, 3.5)^a^	20.8, CH_2_	1.45, m^a^
		1.32, dt (13.8, 4.4)^a^		1.23, m
9	30.6, CH_2_	1.42, m^a^	24.3, CH_2_	1.67, m
		1.29, m^a^		1.31, m^a^
10	33.7, CH	2.54, m	31.2, CH	2.19, m^a^
11	171.7, C		172.3, C	
12	98.0, CH	6.52, s	95.6, CH	5.94, s
13	161.3, C		160.7, C	
14	95.2, C		95.0, C	
15	164.2, C		163.9, C	
16	16.2, CH_3_	0.92, d (6.9)	18.8, CH_3_	1.07, d (7.3)
17	190.6, CH	9.88, d (0.7)	191.5, CH	9.98, d (0.7)
18‐NH	—	10.76, s‐broad	—	10.84, s
19	46.1, CH_2_	3.66, q (5.4)	45.0, CH_2_	3.51, q (5.8)
20	61.2, CH_2_	3.80, t (5.0)	61.3, CH_2_	3.88, t (5.3)
3‐OH	—	4.69, d (4.5)	—	—
4‐OH	—	4.20, t (5.3)	—	—

*Note*: NMR assignments were determined on the basis of analysis of 1D and 2D NMR data, including ^1^H–^1^H COSY and ^1^H–^13^C HSQC and HMBC correlations.

^a^Overlapped signal.

**FIGURE 2:**
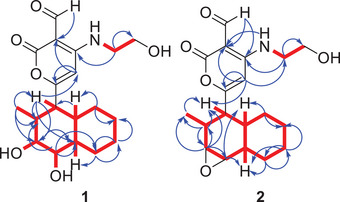
Key ^1^H–^1^H COSY (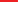) and HMBC (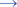) correlations of compounds **1** and **2**.

Analysis of the ^13^C and 2D NMR spectra (HMBC, COSY; Figure 2) indicated that compound **1** is a solanapyrone analogue, distinguished by hydroxy substitutions at C‐3 and C‐4 positions, typically involved in a double bond in related structures. The chemical shifts of the decalin moiety were assigned on the basis of key HMBC and COSY correlations. The signal for C‐2, which appeared weakly in the HSQC spectrum, was observed at *δ*_C_ 33.7. The corresponding proton appeared as a broad multiplet, and its assignment was supported by HMBC correlations with H‐1, H_3_‐16, and H‐4, as well as a COSY correlation between the proton at *δ*_H_ 2.44 and the signals for H‐1 and H_3_‐16.

**FIGURE 3:**
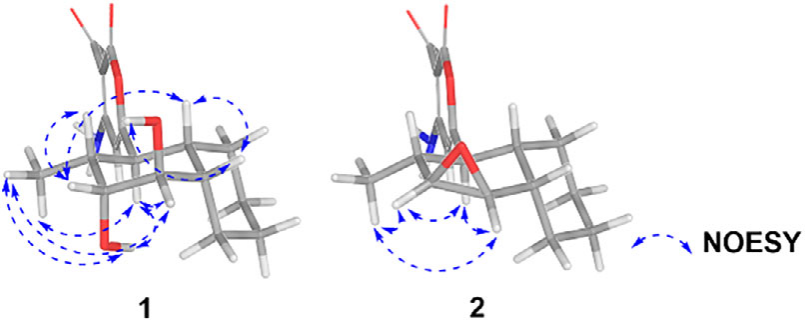
Key ^1^H–^1^H NOESY correlations of compounds **1** and **2**, depicted using geometry‐optimized molecular models. The flexible side chain (–CH_2_CH_2_OH) has been omitted for clarity.

The relative configuration of compound **1** was determined through analysis of *J*‐coupling constants and NOE cross‐peaks. H‐1 appeared as a doublet of doublets (*J* = 11.6, 11.6 Hz), suggesting an *anti*‐orientation with both H‐2 and H‐10. Broad signals were observed for H‐3 and H‐4, likely indicating rapid relaxation caused by electronic effects from the adjacent oxygen atom. H‐3 appeared as a broad triplet, with a coupling constant of *J* = 3.5 Hz, suggesting a dihedral angle of about 60° between H‐3 and H‐2. In contrast, H‐4 exhibited a broad doublet of doublets with a coupling constant of *J* = 1.8, 1.8 Hz, consistent with near‐orthogonal relationships between H‐4/H‐5 and H‐4/H‐3. NOE correlations supported this interpretation, showing interactions between H‐1/H_3_‐16, H‐1/3‐OH, H‐1/H‐4, 3‐OH/H_3_‐16, H‐3/H‐10, 4‐OH/H‐5, and H‐5/H‐10 and H‐4/H_3_‐16 (Figure 3).

These data proposed an *exo*‐decalin as a backbone, with hydroxy group configurations consistent with those reported for sphasolanapyrone A, a recently described compound, which bears a C‐3/C‐4 vicinal diol moiety [8]. However, in contrast to sphasolanapyrone A, this compound features a two‐methylene chain and a primary alcohol, resembling other solanapyrones such as solanapyrone C [9].

For nigrosporapyrone F (**2**), the molecular formula was determined to be C_19_H_25_NO_5_ on the basis of *m/z* 346.1650 [M ‐ H]^‐^ (calcd for C_19_H_24_NO_5_, 346.1649, *Δ* + 0.3 ppm), indicating eight degrees of unsaturation. NMR spectroscopic analysis revealed signals similar to those of compound **1** (Table 1); however, the oxygenated methine signals observed in **1** were replaced by two upfield‐shifted signals, corresponding to oxygenated methine protons at *δ*_H_ 3.00 (H‐4) and *δ*_H_ 2.90 (H‐3).

To rationalize the eight degrees of unsaturation along with the observed NMR spectroscopic features, the presence of an epoxide group between C‐3 and C‐4 was proposed. On the basis of the coupling constants, a *cis* configuration was inferred between H‐3 and H‐4, consistent with the observed *J* = 3.9 Hz. Additionally, the appearance of H‐3 as a doublet suggests a close to 90° angle with H‐2. In turn, H‐4 appeared as a doublet of doublets (*J* = 3.9, 2.0 Hz), where the smaller coupling constant is attributed to H‐5. Furthermore, NOE cross‐peaks between H‐1/H‐3, H‐1/H‐4, H‐3/H_3_‐16, and H‐4/H_3_‐16 provided key spatial information to establish the relative configuration of the molecule. In contrast, correlations involving H‐1, H‐2, H‐5, and H‐10 could not be reliably evaluated due to signal overlapping in the range of 2.20–2.30 ppm (Figure 3). Nigrosporapyrone F (**2**) is the first solanapyrone reported to feature an epoxide group in place of the double bond between positions 3 and 4.

**FIGURE 4:**
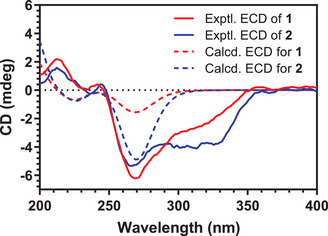
Experimental (solid line) and calculated (dashed line) ECD curve of compounds **1** (red) and **2** (blue). ECD, electronic circular dichroism.

On the basis of the relative configuration of the six stereogenic centers, unambiguously established by analysis of key NOE correlations—the absolute configurations of compounds **1** and **2** were determined by comparison of the experimental and calculated ECD spectra (Figure 4). Experimentally, both compounds exhibited positive Cotton effects around 213 nm and near 244 nm. Negative Cotton effects were observed in the 260–330 nm region for both compounds, with pronounced minima at 270 nm for compound **1**, and at 264, 296, and 326 nm for compound **2**.

Notably, compound **1** and sphasolanapyrone A keep the same core structure with respect to the configuration of the six stereogenic centers. The ECD spectrum of sphasolanapyrone A, with an absolute configuration of (1*S*,2*R*,3*R*,4*R*,5*S*,10*R*), recently reported by Wang and colleagues [8], exhibits two positive Cotton effects below 250 nm and a series of negative Cotton effects between 260 and 350 nm. This pattern closely resembles both the calculated and experimental ECD spectra of compounds **1** and **2**. On the basis of this similarity, and considering the conserved biosynthetic origin of these products, the absolute configurations 1*S*,2*R*,3*R*,4*R*,5*S*,10*R* and 1*S*,2*R*,3*S*,4*R*,5*S*,10*R* were proposed for compounds **1** and **2**, respectively. Notably, the only difference between the two compounds lies in the stereochemistry at C‐3, which is influenced by the presence of a *cis*‐epoxide in compound **2**. Under acidic aqueous conditions, the epoxide can undergo ring opening to generate **1**. In this reaction, protonation of the epoxide makes it a good leaving group, allowing water to attack the more substituted carbon. This nucleophilic attack breaks the C–O bond and leads to inversion of configuration at this asymmetric center [10].

Both compounds feature an *exo*‐fused decalin system, representing the thermodynamically and enzymatically favored product of the Diels–Alderase‐catalyzed intramolecular [4 + 2] cycloaddition characteristic of this molecular family [4, 11]. Consistent with this observation, all compounds isolated in this study retain the *exo*‐decalin framework and were identified on the basis of spectroscopic data reported in the literature. Among them, two were recently described as new natural products: sphasolanapyrone F (**3**) [8] and solanapyrone W (**4**) [12]. Additionally, compounds **5–9** were identified as nigrosporapyrone B (**5**), nigrosporapyrone C (**6**) [13], solanapyrone G (**7**) [14], solanapyrone B (**8**), and solanapyrone C (**9**) [9].

All isolated compounds were evaluated for their ability to inhibit the activity of human long‐chain protein tyrosine phosphatase 1B (*h*PTP1B_1–400_) [15], considering the bioactivity displayed by the extract according to Martínez‐Aldino [5]. PTP1B is a prototypical intracellular non‐receptor tyrosine phosphatase that plays a key central role in numerous signaling cascades related to insulin metabolism and oncogenesis [16]. The *h*PTP1B_1–400_ variant used in this evaluation comprises the first two of its three domains: an N‐terminal catalytic domain (residues 1–300) and a regulatory domain (residues 301–400). The third domain, not included in this construct, corresponds to the C‐terminal domain (residues 401–435). A well‐characterized natural product with known inhibitory activity was used as a positive control (duclauxin at 20 µM) [17, 18]. No significant inhibition was observed, as only sphasolanapyrone F (**3**) and solanapyrone B (**7**) exhibited inhibitory effects at 100 µM, reducing enzymatic activity by 37% and 33%, respectively (Supporting Information section, Figure S29). Moreover, inhibition [5] against the multidrug‐resistant *A. baumannii* strain A564 [19] at 200 µg/mL was also tested for the two major compounds. Of these, product **9** noted for low inhibition as 31.6% (Supporting Information section, Table S1). Given the bioactivity results for both assays, the extract of *Nigrospora* sp. IQ‐064 may contain non‐isolated metabolites that, together with those reported here, are responsible for its effect.

This family of compounds have been isolated from endophytic or marine fungi, such as those belonging to the genera *Alternaria* [9, 11, 20], *Ascochita* (phylum Dothideomycete) [21], *Peroneutypa* [22], and *Nigrospora* (phylum Sordariomycetes) [8, 12, 13, 23], among other unidentified filamentous fungi [14, 24]. Previous studies have reported various biological activities for this chemical family, including mild antifungal [23, 24] and antimicrobial [8, 13, 20, 24] effects, as well as antialgae toxicity [14]. Notably, they are well documented as phytotoxins [9, 11, 21], and more recently, their potential neuroprotective properties have also been explored [12]. The moderate activities observed across different targets suggest that their most promising bioactivity may yet be uncovered. Given the relevance of this chemical scaffold, an in silico ADME and drug‐likeness analysis using the SwissADME platform was conducted (http://www.swissadme.ch/, Supporting Information section) [25], along with a PASS (prediction of activity spectra for substances) analysis (http://www.way2drug.com/passonline; Supporting Information section) [26], to support and justify further investigations into their biological potential.

All compounds analyzed (**1–9**) exhibit physicochemical properties within commonly accepted ranges for orally bioavailable drugs. Lipophilicity, as estimated by the consensus Log *P*, ranged from 1.26 to 3.10, suggesting a balanced profile between solubility and membrane permeability. Predicted aqueous solubility (on the basis of ESOL, Ali, and Silicos‐IT models) classified all compounds as soluble or moderately soluble, with sphasolanapyrone F (**3**) showing the lowest solubility across models. All compounds comply with the major drug‐likeness filters, including Lipinski, Veber, Ghose, Egan, and Muegge rules, and present acceptable bioavailability scores (0.55 or 0.56). No PAINS structural alerts were detected, and the number of Brenk alerts was minimal (1–3 per compound). Synthetic accessibility ranged from 4.63 to 5.21, indicating moderate synthetic feasibility. Notably, compounds **2** and **4–6** fulfill the criteria for lead‐likeness. In fact, several synthetic approaches have been describes for members of this molecular family. A comprehensive review on this topic can be found in a recent publication by Berlinck and Skellam [4].

The PASS online resource [26] is a tool designed to predict the biological activity spectra of organic compounds on the basis of structure‐activity relationship (SAR) analysis. Its prediction model relies on Bayesian statistics and is trained on a large dataset of known bioactive compounds. In this analysis, only biological activities with a probability of activity (Pa) ≥ 0.7 were considered (Table S3, Supporting Information section), as this threshold is generally associated with a reasonable likelihood of experimental validation. The most consistently predicted activity was CDP‐glycerol glycerophosphotransferase inhibition, a key enzyme involved in the biosynthesis of teichoic acids, essential components of the Gram‐positive bacterial cell wall [27, 28]. Seven of the compounds—excluding **3** and **8**—showed Pa values between 0.72 and 0.80. This predicted activity may represent a potential mode of action underlying the previously observed antibacterial effects against Gram‐positive bacteria, such as *Staphylococcus aureus* and MRSA [13, 20, 24]. Likewise, most compounds showed moderate probabilities as phosphatase inhibitors, with Pa values up to 0.73. The prediction included approximately 72 different phosphatases, of which around 30 were various types of protein tyrosine phosphatases. Notably, the PTP1B enzyme, which was used in our biological assays, is not among the targets represented in the PASS database (https://www.way2drug.com/dr/regnet.php).

This could help explain the modest biological activity observed in this study. Moreover, these molecules may serve as inspiration for developing new analogs targeting various classes of phosphatases, which are relevant in cancer [29], metabolic [30], and neurodegenerative disorders [31].

Together with the SwissADME analysis, these results support the notion that this chemical scaffold is pharmacologically versatile and warrant further exploration of alternative bioactivities within this compound family. To ensure sufficient quantities of molecules from this class for downstream pharmacological studies, several synthetic routes have been documented [4]. In addition, our group recently developed a fungal culture methodology employing DMSO as an epigenetic modifier, which significantly enhances the biosynthesis of secondary metabolites. If necessary, a similar strategy could be applied to scale up production of the target compounds [32].

## 3 Conclusions

The influence of two different cultivation substrates on the metabolic profile of *Nigrospora* sp. IQ‐064 was analyzed, revealing that the Cheerios‐based medium produced the most chemically diverse extract. A classical chemical investigation of this extract led to the isolation of two new oxygenated solanapyrone analogs, along with seven known compounds. Notably, all isolated solanapyrones corresponded to the *exo*‐decalin type, which appears to be the most favorable form under the cultivation conditions employed.

All compounds were evaluated for their ability to inhibit PTP1B, but none exhibited significant inhibitory activity. However, in silico predictions, including ADME profiling and PASS analysis, suggest that some of these metabolites may be worth exploring further from a pharmacological perspective, as four compounds showed lead‐like properties. In particular, the predicted inhibition of CDP‐glycerol glycerophosphotransferase based on PASS analysis, an enzyme involved in the biosynthesis of teichoic acids in Gram‐positive bacteria, may provide insights into the previously reported antibacterial activity of this compound class. Moreover, moderate probabilities of inhibition were observed for other phosphatases (including tyrosine phosphatases distinct from PTP1B), which may warrant further experimental investigation in future studies.

## 4 Experimental Section

### 4.1 General Experimental Procedures

Chromatographic techniques were conducted using the following instruments. For primary fractionation, flash chromatography was performed on a Pure C‐810 flash chromatograph (Büchi) equipped with photodiode array UV detection (PDA), which scanned between 230 and 500 nm. Fraction analysis and compound isolation were conducted using analytical and semipreparative HPLC, respectively, on a Waters chromatograph equipped with a PDA detector (model 2998) coupled to an evaporative light‐scattering detector (ELSD; model 2424). For analytical and semipreparative analyses, an octadecyl column Gemini‐NX with a particle size of 5 µm was used (4.6 mm × 250.0 mm for analytical and 10.0 mm × 250.0 mm for semipreparative). Data acquisition and management were performed using Empower 3 software. NMR data for ^1^H, ^13^C, and 2D were recorded in CDCl_3_ or Me_2_CO‐*d*_6_ on a Bruker Ascend III 700 MHz spectrometer equipped with a cryoprobe, operating at 700 MHz for ^1^H and 175 MHz for ^13^C or Bruker Ascend 500 MHz equipped with a BBFO probe, operating at 500 MHz for ^1^H and 125 MHz for ^13^C (LURMN, IQ‐UNAM). Chemical shifts are presented in parts per million relative to the internal standard solvent resonance. Mass spectra of extracts and pure compounds were obtained using a Q Exactive Plus Hybrid Quadrupole‐Orbitrap (Thermo Fisher Scientific) equipped with an Acquity ultra‐efficient liquid chromatography (UPLC) system (Waters), consisting of a BEH C18 column (1.7 µm, 130 Å, 2.1 × 50 mm). The elution system used a gradient of methanol and water acid (HCOOH 0.1%), ranging from 15% to 100% methanol in 8 min. A Jeol AccuTOF JMS‐T100LC mass spectrometer coupled with DART detection (HR‐DART) was also used.

Optical rotation and ECD were measured in methanol using a Jasco J‐1500 polarimeter and a Jasco J‐1500 spectropolarimeter, respectively.

### 4.2 Metabolomic Analysis

The chemical diversity of fungi on two substrates (Cheerios and rice) was analyzed by classical molecular networks with the online platform GNPS Molecular Networking [33] and from its chemical profile obtained by high‐resolution tandem mass spectrometry (HPLC‐HRESI‐QTOF) available in the MassIVE repository MSV000091237 [5]. The construction of molecular networks was performed according to previous described parameters [34, 35]. Promising identification of known compounds started by manually comparing spectrometric information (MS1) against metabolites reported for the genus *Nigrospora* indexed by LOTUS through the National Center for Biotechnology Information (NCBI; 64 compounds), the Natural Products Atlas (64 entries), Dictionary of Natural Products (123 entries), and selected bibliography on the constituents of the genus [2, 7]. Identifications were considered if the molecular ion mass match had an error <10 ppm and at least three fragments were shared with information published in peer‐reviewed journals.

### 4.3 Fungal Strain Isolation

Strain *Nigrospora* sp. IQ‐064 (NCBI accession number: OQ349253) was isolated from the bark of black mangrove (*A. germinans*) and described in a previous publication [5].

### 4.4 Extraction and Isolation

For the small‐scale extraction, small plugs of *Nigrospora* sp. IQ‐064 grown on potato dextrose agar were inoculated into 10 mL of potato dextrose broth and incubated at room temperature for 5 days on a rotary shaker. The resulting cultures were then transferred to 250 mL Erlenmeyer flasks containing two different solid media: one consisting of 10 g of autoclaved Cheerios and the other of 12 g of autoclaved rice with 24 mL of distilled water.

For the scaled‐up organic extract, the same methodology was employed using only Cheerios cereal as the substrate, in 20 replicates. All cultures (both small‐scale and scale‐up) were maintained at room temperature for 21 days and subsequently macerated overnight with a 1:1 mixture of CH_2_Cl_2_–MeOH. The biomass was removed by filtration, and the filtrate was extracted with distilled water. The organic layer was separated using a separatory funnel and dried *in vacuo*. The resulting crude extracts were defatted with a 2:1:1 mixture of hexanes–MeCN–MeOH. The polar fraction was separated and concentrated, yielding a sufficient amount for metabolomic assays in the small‐scale extracts and 1.77 g of organic extract from the scaled‐up culture.

The scaled‐up extract was adsorbed onto Celite 545 and fractionated by flash chromatography on a 24 g silica gel column, eluted with a gradient solvent system of hexanes–EtOAc–MeOH at a flow rate of 15 mL/min. A total of 96 column volumes were collected, and fractions were pooled on the basis of UV detection signals. Fourteen fractions (F1–F14) were obtained, and the isolation of pure compounds is described below.

F5 (65.0 mg of 78.5 mg) was subjected to semi‐preparative HPLC using a gradient system from 30:70 to 100:0 of MeCN‐0.1% aqueous formic acid over 30 min at a flow rate of 4.7 mL/min, yielding **7** (4.2 mg, *t_R_* = 14.2 min).

F6 (107.0 mg of 148.5 mg) was subjected to semi‐preparative HPLC using a gradient system from 30:70 to 100:0 of MeCN‐0.1% aqueous formic acid over 30 min at a flow rate of 4.7 mL/min, yielding **4** (2.2 mg, *t_R_* = 6.6 min), **8** (2.6 mg, *t_R_* = 14.8 min), and **9** (23.6 mg, *t_R_* = 15.3 min).

F8 (70.0 of 90.3 mg) was subjected to semi‐preparative HPLC using a gradient system from 25:85 to 100:0 of MeCN‐0.1% aqueous formic acid over 30 min at a flow rate of 4.7 mL/min, yielding **5** (1.1 mg, *t_R_* = 6.8 min), **4** (15.1 mg, *t_R_* = 8.7 min), **2** (2.0 mg, *t_R_* = 11.4 min), **1** (1.8 mg, *t_R_* = 13.2 min), and **3** (1.2 mg, *t_R_* = 15.9 min).

F10 (48.0 of 95.6 mg) was subjected to semi‐preparative HPLC using a gradient system from 20:80 to 100:0 of MeCN‐0.1% aqueous formic acid over 30 min at a flow rate of 4.7 mL/min, yielding **5** (1.5 mg, *t_R_* = 8.9 min) and **6** (1.5 mg, *t_R_* = 9.8 min).

**Nigrosporapyrone E (1)**. White solid. [*α*]^25^_D_ −6.67 (*c* 0.12 MeOH); ECD (MeOH, *c* 1.2 mg/mL) *λ*_max_ (Δ*ε*) 213 (2.1), 242 (0.4), 270 (−6.2), 320 (−2.3), 358 (0.3); ^1^H NMR (CDCl_3_, 700 MHz) and ^13^C NMR (CDCl_3_, 175 MHz) data, see Table 1; HR‐ESI‐MS *m/z* 364.1758 [M‐H]^−^ (calcd for C_19_H_26_NO_6_, 364.1755, *Δ* + 0.9 ppm).

**Nigrosporapyrone F (2)**. Colorless vitreous solid. [*α*]^25^_D_ −15.45 (*c* 0.11 MeOH); ECD (MeOH, *c* 1.1 mg/mL) *λ*_max_ (Δ*ε*) 212 (1.6), 235 (−0.2), 244 (0.4), 264 (−5.3), 296 (−4.0), 326 (−3.7), 366 (−0.2); ^1^H NMR (CDCl_3_, 700 MHz) and ^13^C NMR (CDCl_3_, 175 MHz) data, see Table 1; HR‐ESI‐MS *m/z* 346.1649 [M ‐ H]^‐^ (calcd for C_19_H_24_NO_5_, 346.1649, *Δ* + 0.3 ppm).

### 4.5 ECD Calculations of Compounds 1 and 2

The truncated models of compounds **1** and **2**, in which the NHCH_2_CH_2_OH fragment was replaced with an NH_2_ group, were optimized using the PM3 semiempirical method in Spartan 10 software. Subsequently, a conformational analysis was performed at the same level of theory to identify and remove redundant conformers. The geometries of the resulting conformers were further optimized in Gaussian 09 using density functional theory (DFT) at the ωB97XD/def2‐SVP level in MeOH. ECD spectra were simulated from the excited‐state calculations using a Lorentz/Gaussian function, according to the following equation:

(1) $$\Delta \varepsilon \left(E\right)=\frac{1}{{2.297_{x10}}^{-39}}.\frac{1}{\sigma \sqrt{\pi }}\sum_{k}E_{0k}R_{0k}\exp\left[-{\left\{\frac{E-E_{0k}}{\sigma }\right\}}^{2}\right]$$

where *E*_0k_ and *R*_0k_ are the transition energy and the rotatory strength of the *k*th electronic transition, respectively, and *σ* is half the bandwidth at 1/e peak height. All calculations were performed on the HP Cluster Platform 3000 SL “Miztli,” a parallel supercomputer running a Linux operating system, with 25,312 cores and a total of 15 000 GB of RAM.

### 4.6 Bioassay Procedures

The inhibitory potential of the isolated compounds against hPTP1B_1–400_ was evaluated using an in vitro assay conducted in 96‐well plates. Wild‐type PTP1B_1–400_ containing an N‐terminal 6× His‐tag was cloned, transformed, expressed, and purified as previously described [18]. A well‐characterized inhibitor of fungal origin, duclauxin [17], (20 µM in DMSO) was used as a positive control. The isolated compounds were tested at a plate‐concentration of 100 µM in DMSO. Each compound was incubated at 37°C for 10 min with 5 µL of enzyme stock solution (3 µM) in a buffer composed of 50 mM HEPES, 100 mM NaCl, and 1.5 mM DTT (dithiothreitol) at pH 6.8. After incubation, 10 µL of the phosphatase substrate *para*‐Nitrophenylphosphate (*p*‐NPP) at 30 mM in buffer solution was added, followed by an additional 20 min incubation at 37°C. Absorbance was measured at 405 nm. All assays were performed in a final volume of 100 µL per well using a Cytation 5 plate reader (BioTek). Data acquisition and analysis were performed with Gen 5 software. The inhibition percentage was calculated using the following equation.

(2) $$\%\mathrm{In}h=\left(1-\frac{A_{s_{405}}}{A_{b_{405}}}\right)\times 100\%$$

where %In*h* is the percentage of inhibition, *A*_s405_ is the corrected absorbance of the samples under testing (*A*_405_ end − *A*_405_ initial), and *A*_b405_ is the absorbance of the blank (*A*_405_ end blank − *A*_405_ initial blank). The IC_50_ values were calculated by regression analysis using Equation (3), with GraphPad Prism.

(3) $$\%\mathrm{In}h=\frac{A_{100}}{I+{\left(\frac{I}{{\mathrm{IC}}_{50}}\right)}^{S}}$$

Inhibition of bacterial growth of *A. baumannii* strain A564 was determined by a microdilution method in Mueller‐Hinton broth in 96‐well plates, according to Clinical and Laboratory Standards Institute (CLSI) guidelines. Gentamicin and colistin were used as positive controls in sterile deionized at final concentrations of 20 and 64 ppm, respectively. The compounds were dissolved in DMSO at a concentration of 100 ppm. The procedure, incubation conditions and calculation of the percentage inhibition were described by Aguilar‐Colorado et al. [35].

## Author Contributions

**Carlos A. Fajardo‐Hernández**: conceived and designed the experiments, designed and performed the metabolomic analysis, carried out the scale‐up extraction, isolation, and structural characterization of isolated compounds, performed the biological evaluation, and analyzed the ADME‐Tox and PASS predictions. **Ángel Sahid Aguilar‐Colorado**: conceived and designed the experiments, designed and performed the metabolomic analysis, carried out the scale‐up extraction, isolation, and structural characterization of isolated compounds, performed the biological evaluation, and analyzed the ADME‐Tox and PASS predictions. **Leslie Maribel Corona‐Cabello**: designed and performed the metabolomic analysis, carried out the scale‐up extraction, isolation, and structural characterization of isolated compounds, performed the biological evaluation, and analyzed the ADME‐Tox and PASS predictions. **Ingrid Y. Martínez‐Aldino**: collected biological material for the isolation of fungal taxa, conducted small‐scale extractions, and prioritized fungi for chemical investigation. **José Rivera‐Chávez**: conceived and designed the experiments, collected biological material for the isolation of fungal taxa, conducted small‐scale extractions, and prioritized fungi for chemical investigation. All authors contributed to the original draft preparation, review, and editing of the manuscript and have read and approved its final version.

## Conflicts of Interest

The authors declare no conflicts of interest.

## Supporting information




**Supporting File 1**: cbdv70384‐sup‐0001‐SuppMat.pdf

## Data Availability

The data that support the findings of this study are available in the Supporting Information section of this article.
