# Supplementary material for: Oxygenated Solanapyrone Analogs From Nigrospora sp. IQ‐064, a Mangrove Associated Fungus
Source: Chem Biodivers. 2025 Aug 25;22(12):e01912. doi: 10.1002/cbdv.202501912 (PMC12715977; doi:10.1002/cbdv.202501912)
Supplement: Supplementary file 1 — Supporting File 1: cbdv70384‐sup‐0001‐SuppMat.pdf [file CBDV-22-e01912-s001.pdf]

Supporting Information

Oxygenated solanapyrone analogs from *Nigrospora* sp. IQ-064, a mangrove associated fungus

Carlos A. Fajardo-Hernández[a], Ángel Sahid Aguilar-Colorado[a], Leslie Maribel Corona-Cabello[a], Ingrid Yadira Martínez-Aldino[a], and José Rivera-Chávez[a,*]

[a] Instituto de Química, Departamento de Productos Naturales, Universidad Nacional Autónoma de México, Mexico City, Mexico.

, e-mail jrivera@iquimica.unam.mx.

	Page
**Figure S1**. HR-ESI-MS spectrum of **1**	2
**Figure S2**. ^{1}H-NMR spectrum of nigrosporapyrone E (**1**)	2
**Figure S3**. ^{13}C-NMR spectrum of nigrosporapyrone E (**1**)	2
**Figure S4**. HSQC-NMR spectrum of nigrosporapyrone E (**1**)	3
**Figure S5**. HMBC-NMR spectrum of nigrosporapyrone E (**1**)	3
**Figure S6**. COSY-NMR spectrum of nigrosporapyrone E (**1**)	4
**Figure S7**. NOESY-NMR spectrum of nigrosporapyrone E (**1**)	4
**Figure S8**. HR-ESI-MS spectrum of **2**	5
**Figure S9**. ^{1}H-NMR spectrum of nigrosporapyrone F (**2**)	5
**Figure S10**. ^{13}C-NMR spectrum of nigrosporapyrone F (**2**)	5
**Figure S11**. HSQC-NMR spectrum of nigrosporapyrone F (**2**)	6
**Figure S12**. HMBC-NMR spectrum of nigrosporapyrone F (**2**)	6
**Figure S13**. COSY-NMR spectrum of nigrosporapyrone F (**2**)	7
**Figure S14**. NOESY-NMR spectrum of nigrosporapyrone F (**2**)	7
**Figure S15**. ^{1}H-NMR spectrum of sphasolanapyrone F (**3**)	8
**Figure S16**. ^{13}C-NMR spectrum of sphasolanapyrone F (**3**)	8
**Figure S17**. ^{1}H-NMR spectrum of solanapyrone W (**4**)	10
**Figure S18**. ^{13}C-NMR spectrum of solanapyrone W (**4**)	10
**Figure S19**. ^{1}H-NMR spectrum of nigrosporapyrone B (**5**)	11
**Figure S20**. ^{13}C-NMR spectrum of nigrosporapyrone B (**5**)	11
**Figure S21**. ^{1}H-NMR spectrum of nigrosporapyrone C (**6**)	12
**Figure S22**. ^{13}C-NMR spectrum of nigrosporapyrone C (**6**)	12
**Figure S23**. ^{1}H-NMR spectrum of solanapyrone G (**7**)	13
**Figure S24**. ^{13}C-NMR spectrum of solanapyrone G (**7**)	13
**Figure S25**. ^{1}H-NMR spectrum of solanapyrone B (**8**)	14
**Figure S26**. ^{13}C-NMR spectrum of solanapyrone B (**8**)	14
**Figure S27**. ^{1}H-NMR spectrum of solanapyrone C (**9**)	15
**Figure S28**. ^{13}C-NMR spectrum of solanapyrone C (**9**)	15
**Figure S29.** *h*PTP1B$_{1\text{-}400}$ inhibition percentage for compounds **1**–**9**	16
**Table S1.** Inhibitory activity against *Acinetobacter baumannii* strain A564 by some compounds of *Nigrospora* sp. IQ-064.	16
**Table S2.** ADME properties predicted for compounds **1**-**9**	17
**Table S3.** PASS analysis for compounds **1**–**9**	19

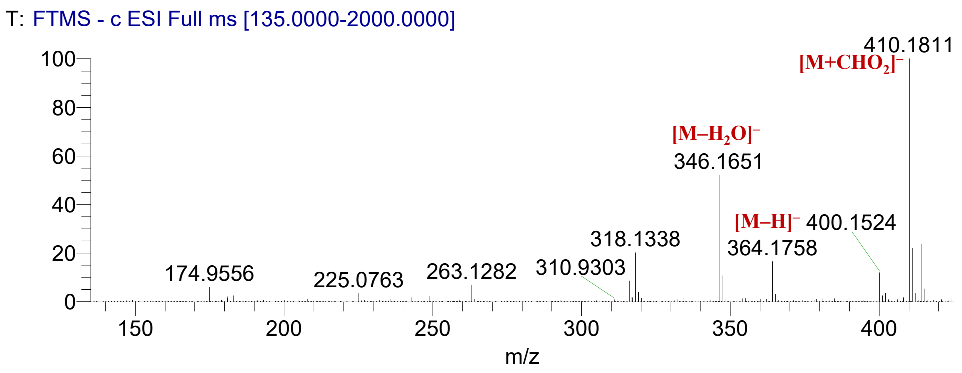

Figure S1. HR-ESI-MS spectrum of nigrosporapyrone E (**1**) *m/z*: 364.1758 $[M–H]^–$ calculated for $C_{19}H_{26}NO_6$ 364.1755, Δ+0.9 ppm.

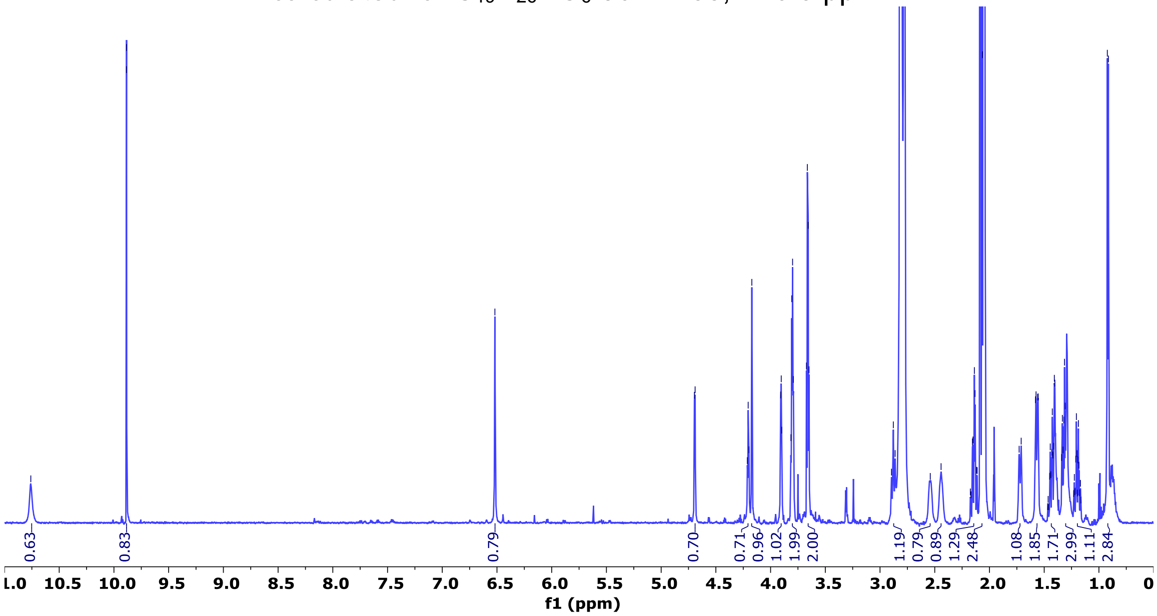

Figure S2. ^{1}H-NMR spectrum of nigrosporapyrone E (**1**) (Acetone-d_6, 700 MHz).

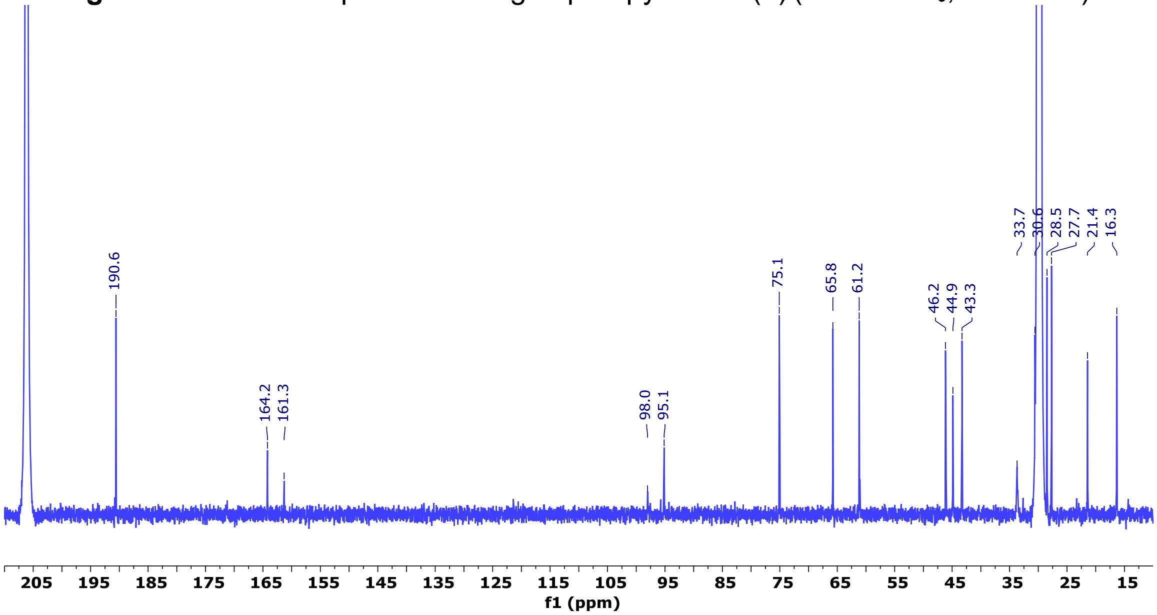

Figure S3. ^{13}C-NMR spectrum of nigrosporapyrone E (**1**) (Acetone-d_6, 175 MHz).

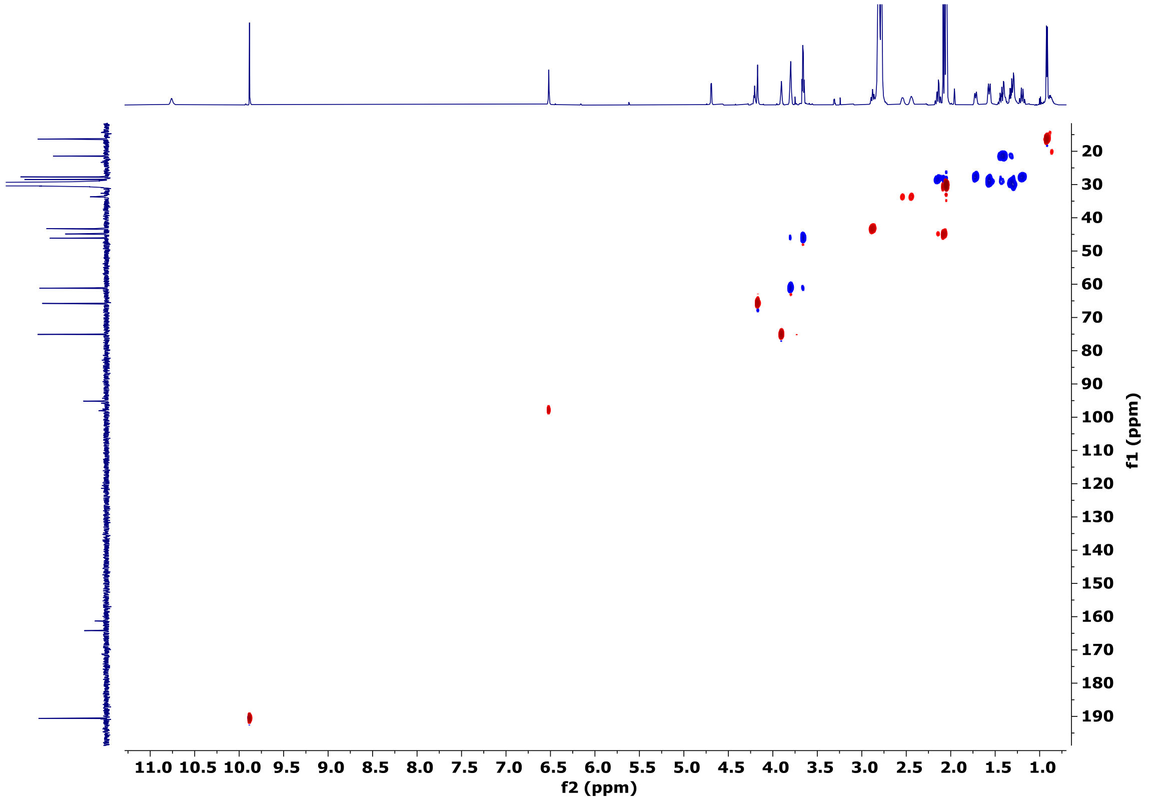

Figure S4. HSQC-NMR spectrum of nigrosporapyrone E (**1**) (Acetone-d_6 700 MHz).

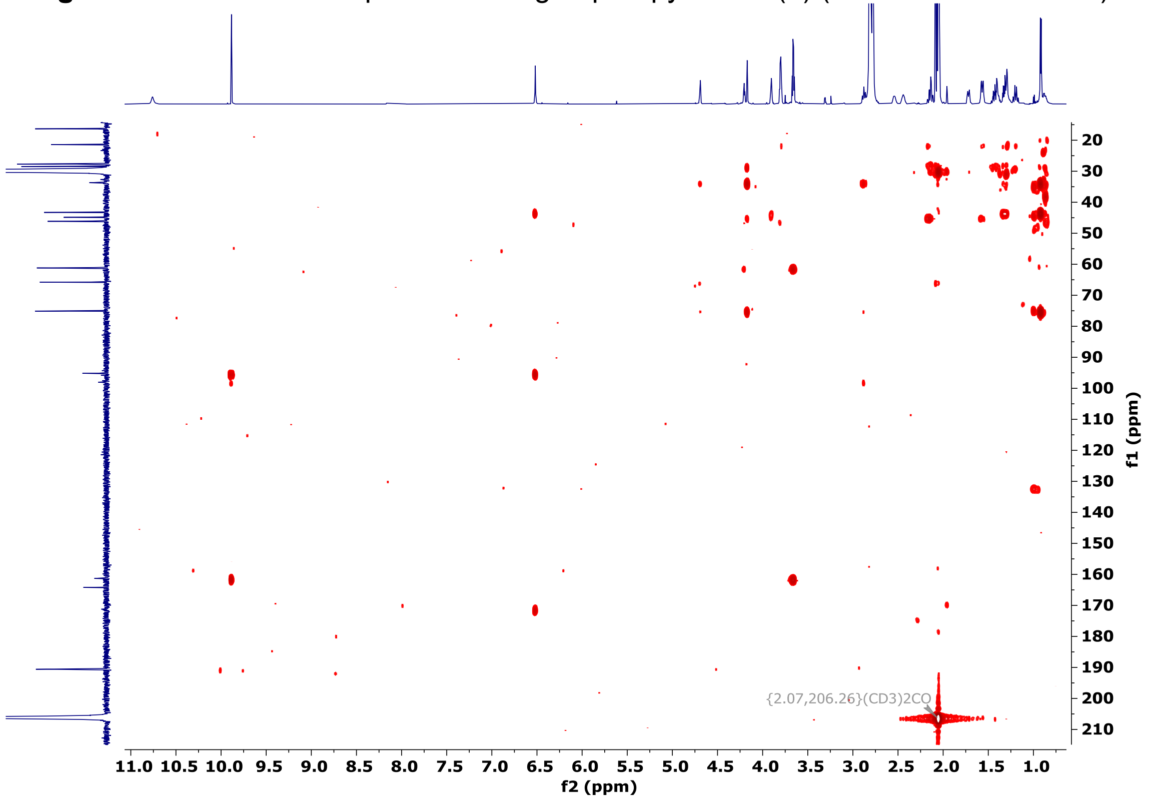

Figure S5. HMBC-NMR spectrum of nigrosporapyrone E (**1**) (Acetone-d_6 700 MHz).

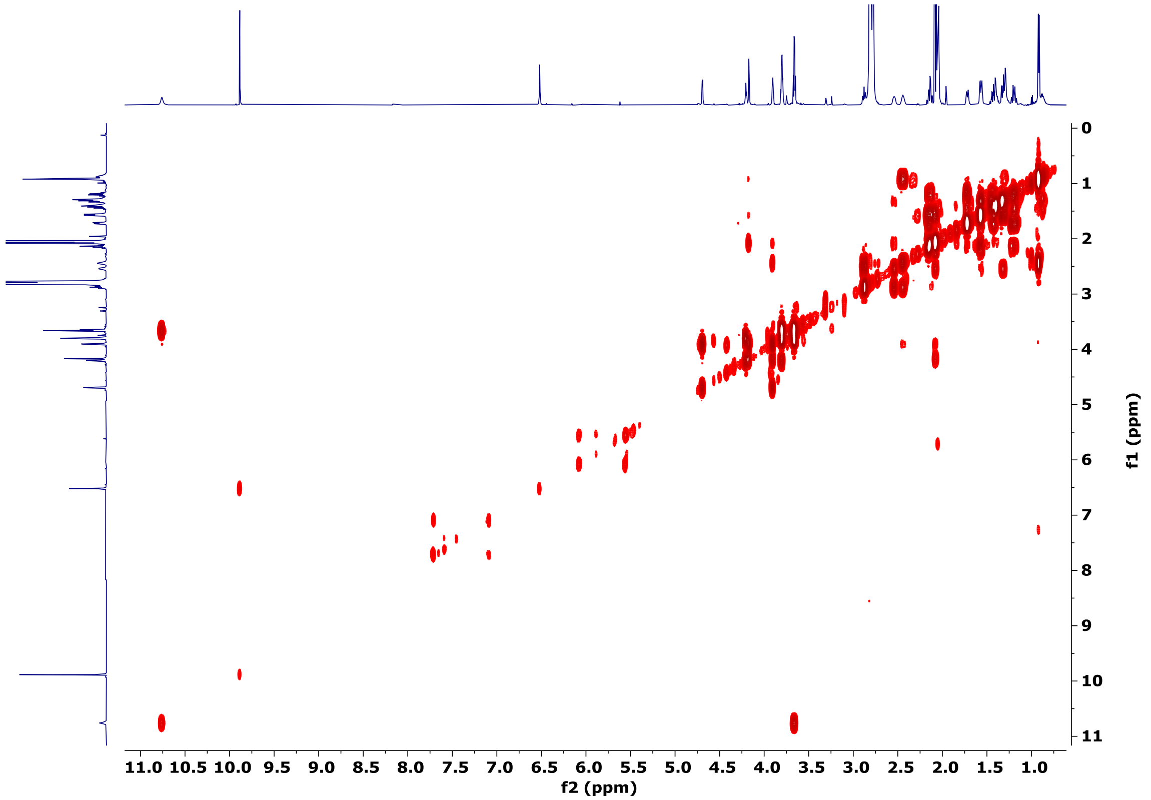

Figure S6. COSY-NMR spectrum of nigrosporapyrone E (**1**) (Acetone-d_6 700 MHz).

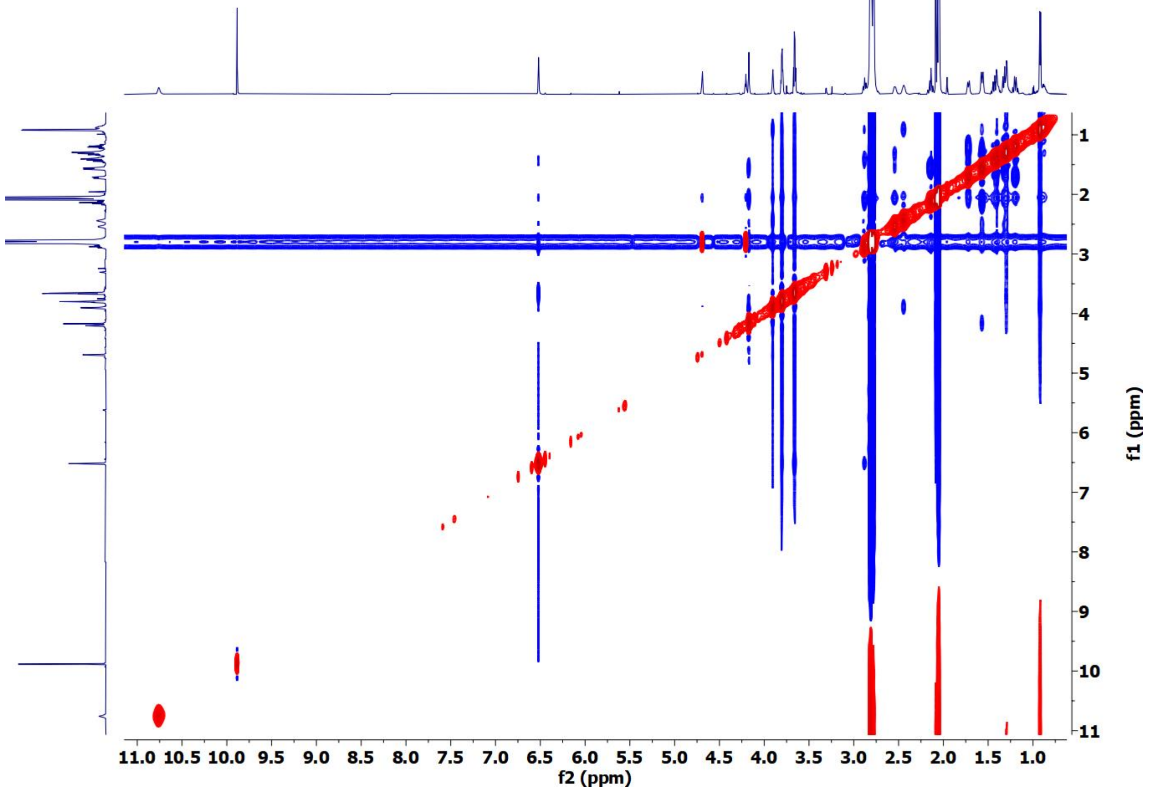

Figure S7. NOESY-NMR spectrum of nigrosporapyrone E (**1**) (Acetone-d_6 700 MHz).

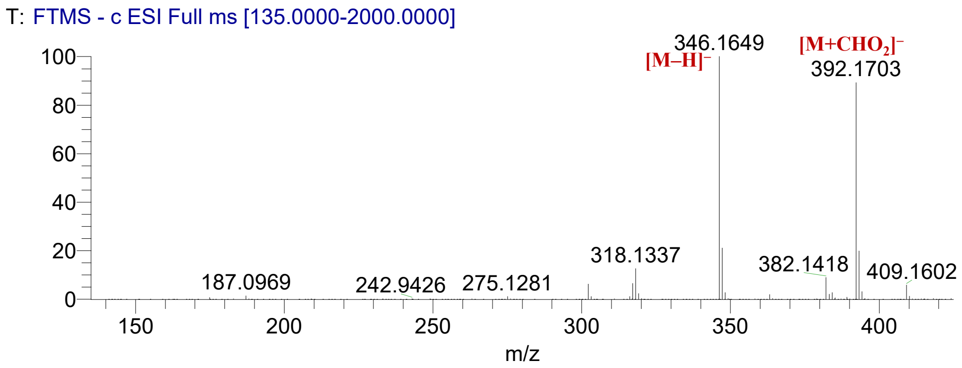

Figure S8. HR-ESI-MS spectrum of nigrosporapyrone F (**2**) *m/z*: 346.1649 [M–H]⁻ calculated for $C_{19}H_{24}NO_5$ 346.1649, Δ 0.0 ppm.

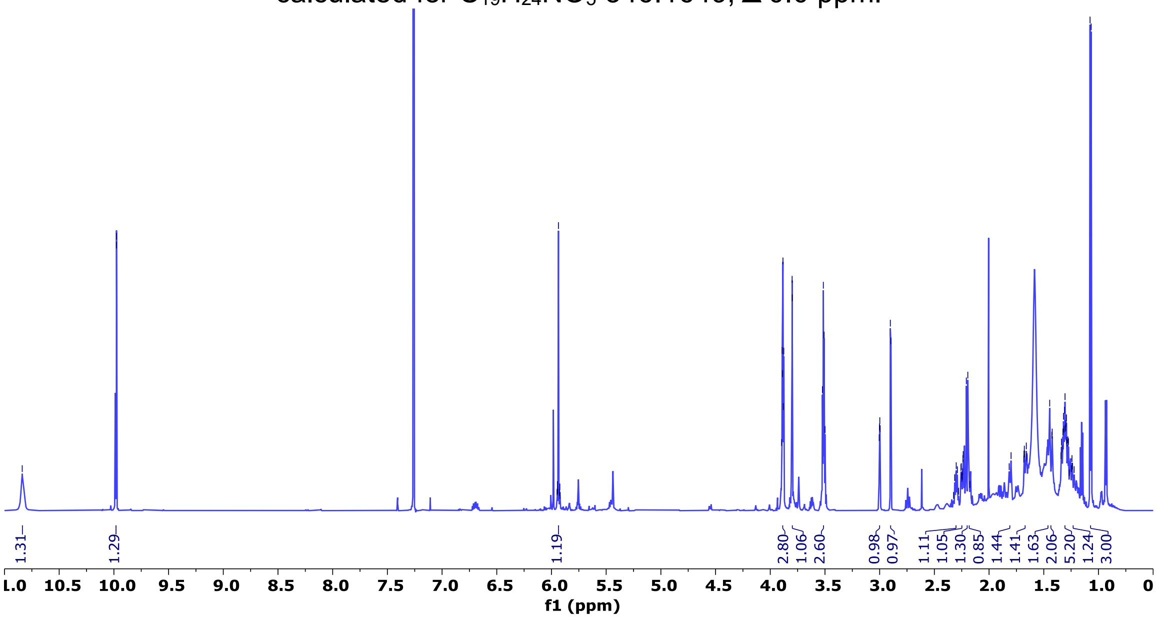

Figure S9. ^{1}H-NMR spectrum of nigrosporapyrone F (**2**) ($CDCl_3$, 700 MHz).

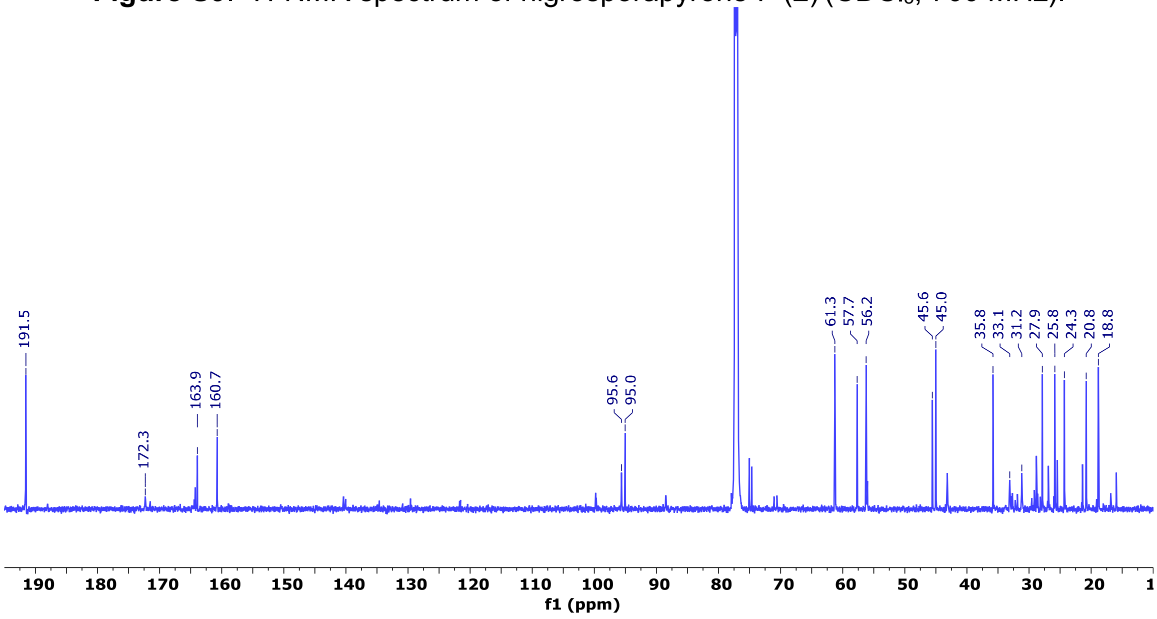

Figure S10. ^{13}C-NMR spectrum of nigrosporapyrone F (**2**) ($CDCl_3$, 175 MHz).

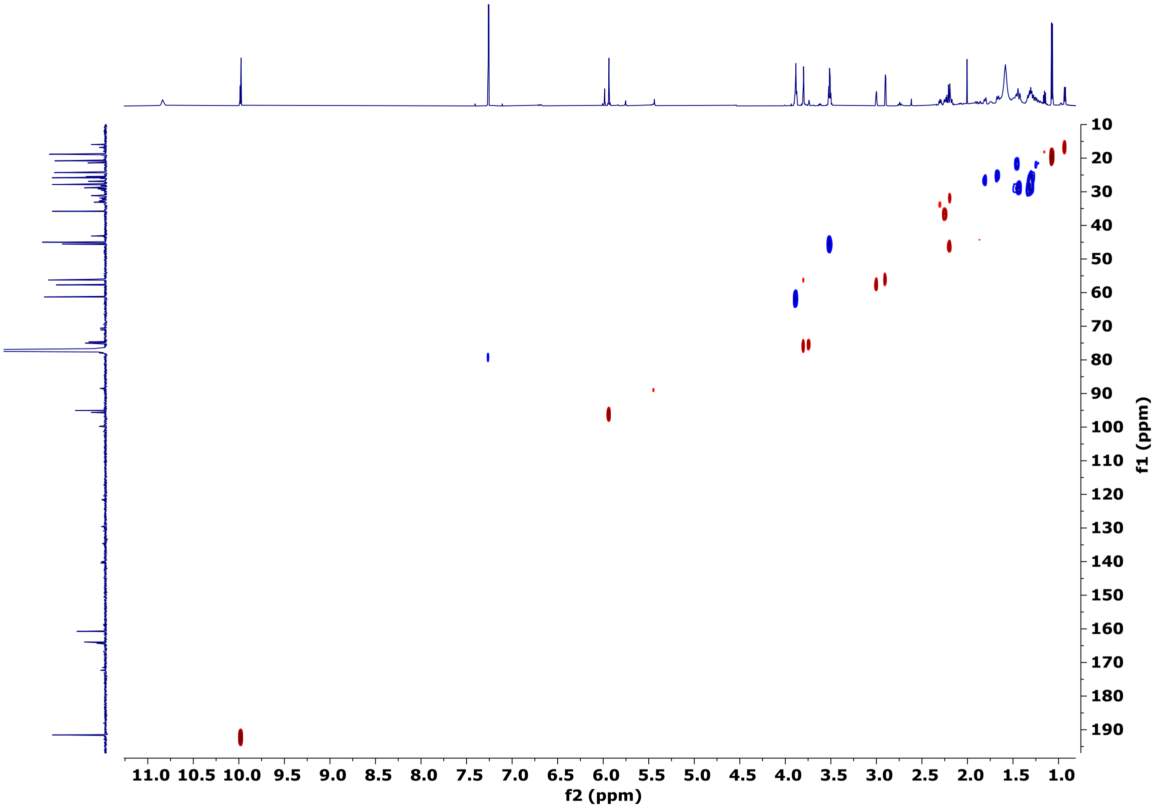

Figure S11. HSQC-NMR spectrum of nigrosporapyrone F (**2**) ($CDCl_3$, 700 MHz).

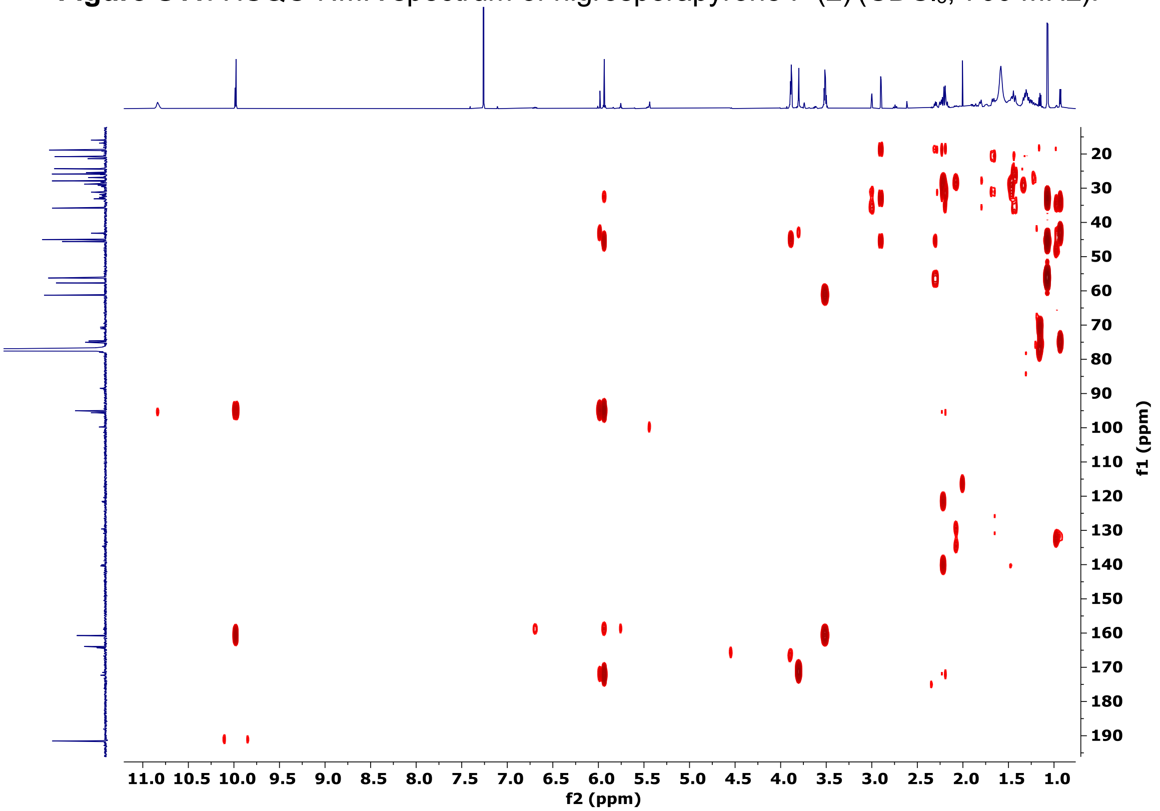

Figure S12. HMBC-NMR spectrum of nigrosporapyrone F (**2**) ($CDCl_3$, 700 MHz).

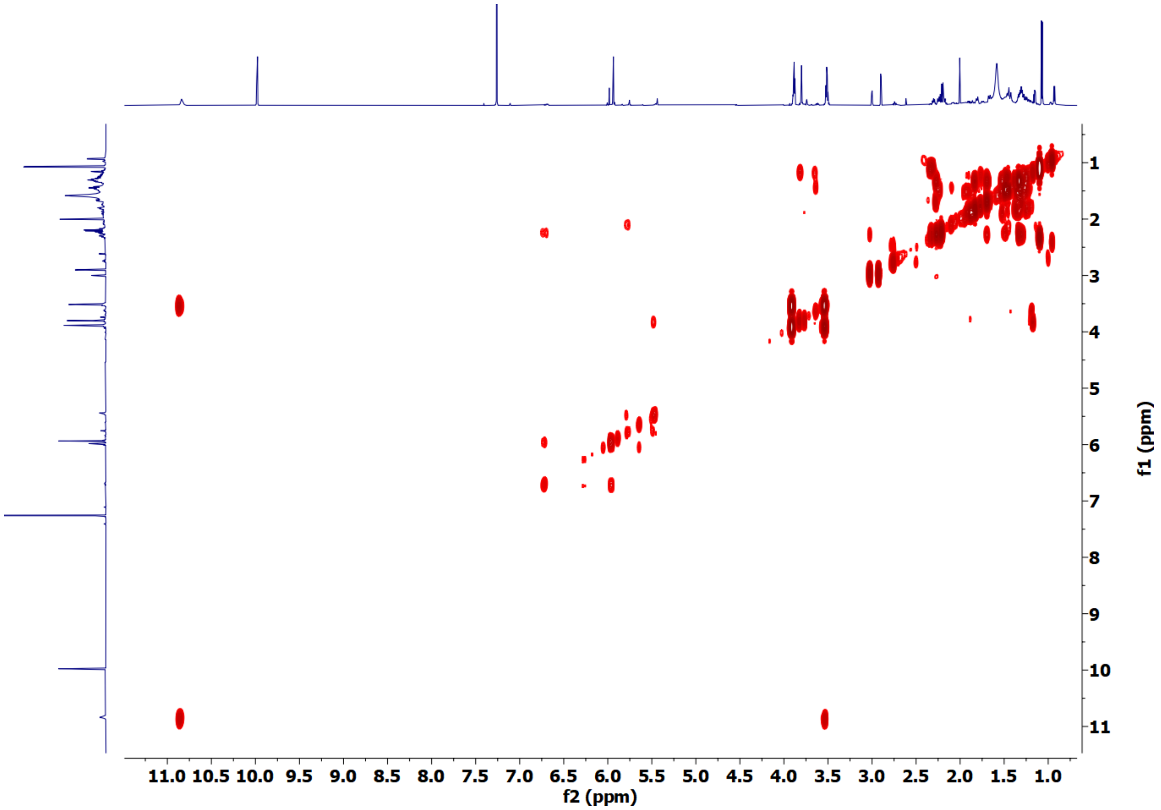

Figure S13. COSY-NMR spectrum of nigrosporapyrone F (**2**) ($CDCl_3$, 700 MHz).

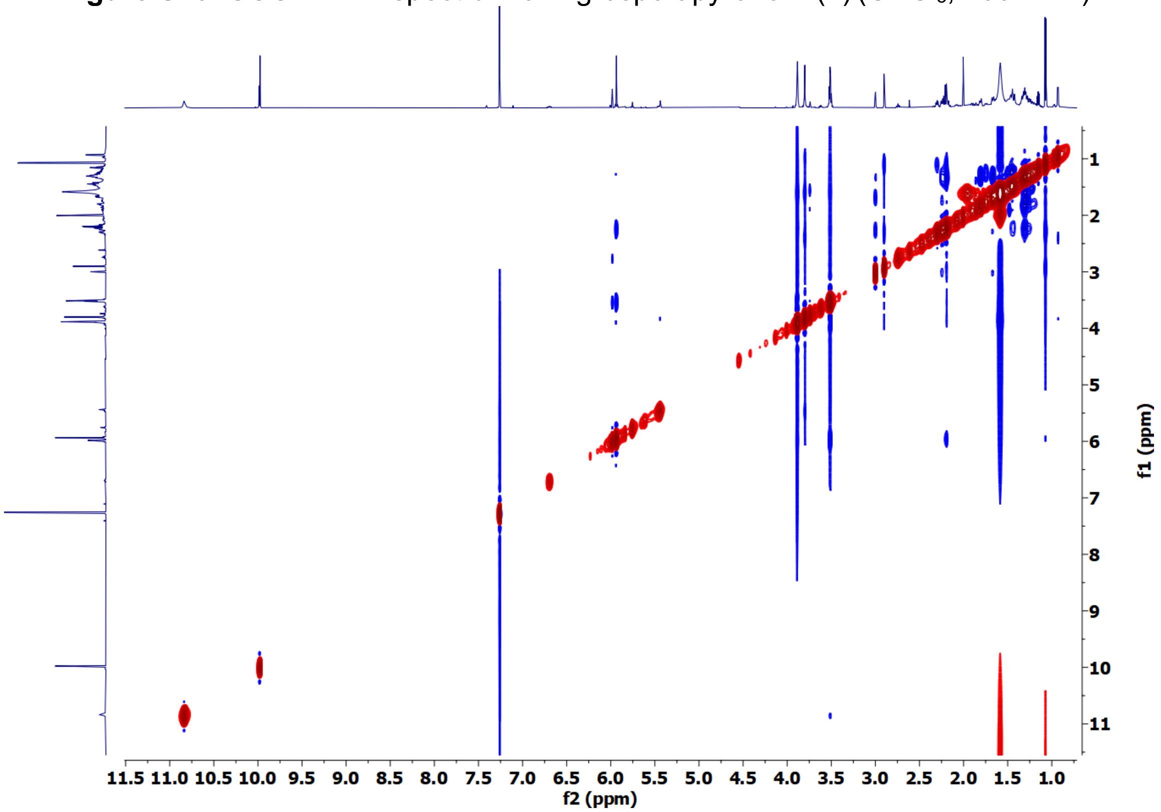

Figure S14. NOESY-NMR spectrum of nigrosporapyrone F (**2**) ($CDCl_3$, 700 MHz).

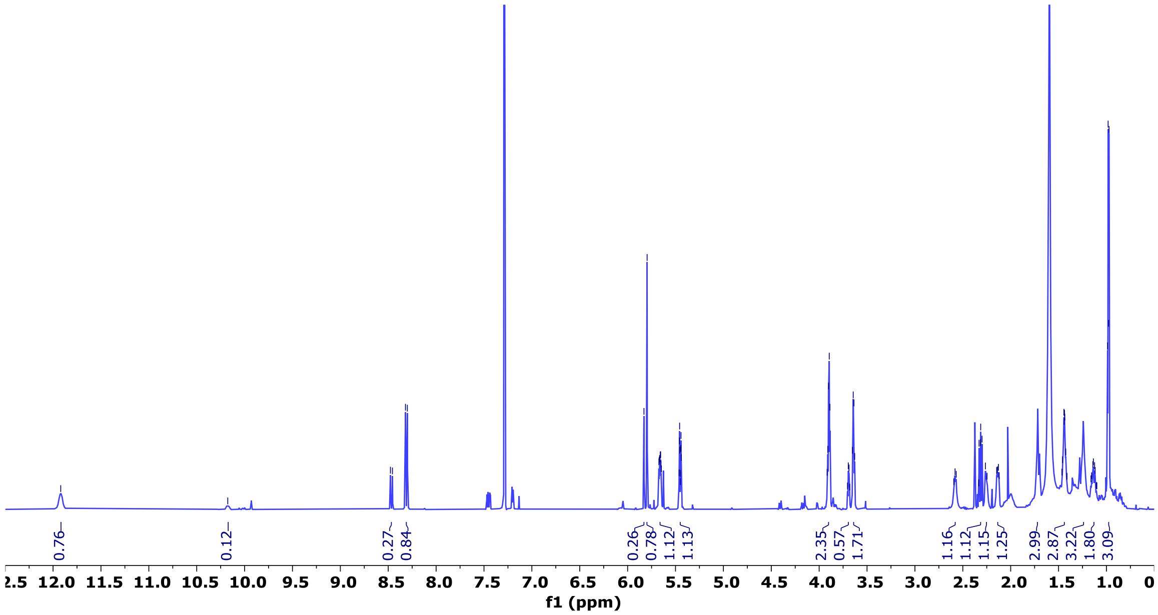

Figure S15. ^{1}H-NMR spectrum of sphasolanapyrone F (**3**) ($CDCl_3$, 700 MHz).

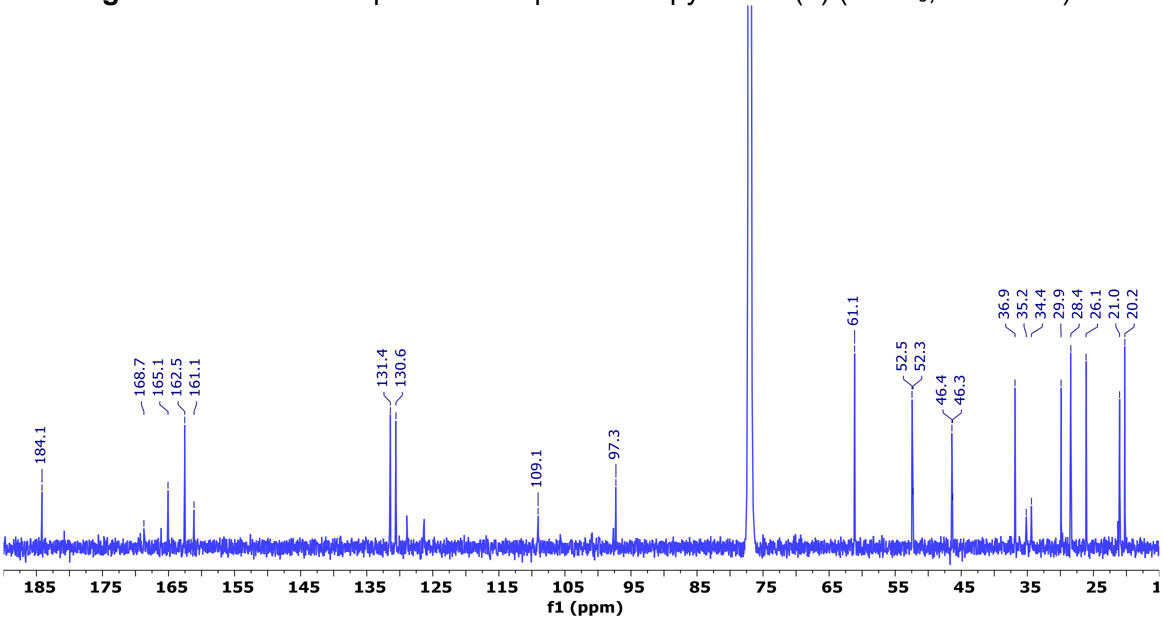

Figure S16. ^{13}C-NMR spectrum of sphasolanapyrone F (**3**) ($CDCl_3$, 175 MHz).

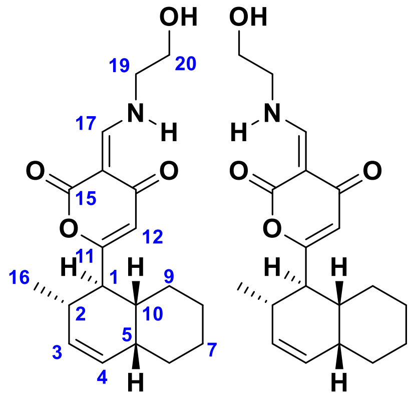

E (major) *Z* (minor)

sphasolanapyrone F (**3**): HR-DART+ *m/z* 332.1846 [M+H]+ calculated for $C_{19}H_{26}NO_4$ 332.1856, Δ –3.1 ppm.

^{1}H NMR (700 MHz, $CDCl_3$): 2(*E*) δ_H 11.92 (s-broad, 1H, NH-18), 8.31 (d, *J* = 13.8 Hz, 1H, H-17), 5.80 (s, 1H, H-12), 5.66 (ddd, *J* = 9.9, 5.0, 2.6 Hz, 1H, H-4), 5.45 (dq, *J* = 9.9, 1.8 Hz, 1H, H-3), 3.90 (dt, *J* = 10.1, 5.0 Hz, 2H, H_2-20), 3.64 (q, *J* = 5.6 Hz, 2H, H_2-19), 2.57 (m, 1H, H-2), 2.31 (dd, *J* = 11.9, 9.8 Hz, 1H, H-1), 2.26 (d-broad, *J* = 11.7 Hz, 1H, H-10), 2.13 (dd, *J* = 12.5, 5.7 Hz, 1H, H-5), 1.70 (m overlapped, 2H, H-6a, H-7a), 1.60 (m, 1H, H-9b), 1.44 (m overlapped, 2H, H-8a, H-9a), 1.24 (m overlapped, 2H, H-7b, H-8b), 1.13 (m, 1H, H-6b), 0.98 (d, *J* = 7.0 Hz, 3H, H_3-16).

2(*Z*) δ_H 10.18 (s-broad, 1H, NH-18), 8.47 (d, *J* = 13.8 Hz, 1H, H-17), 5.83 (s, 1H, H-12), 5.66 (ddd, *J* = 9.9, 5.0, 2.6 Hz, 1H, H-4), 5.45 (dq, *J* = 9.9, 1.8 Hz, 1H, H-3), 3.90 (dt, *J* = 10.1, 5.0 Hz, 2H, H_2-20), 3.69 (q, *J* = 5.5 Hz, 2H, H_2-19), 2.57 (m, 1H, H-2), 2.31 (dd, *J* = 11.9, 9.8 Hz, 1H, H-1), 2.26 (d-broad, *J* = 11.7 Hz, 1H, H-10), 2.13 (dd, *J* = 12.5, 5.7 Hz, 1H, H-5), 1.70 (m overlapped, 2H, H-6a, H-7a), 1.60 (m, 1H, H-9b), 1.44 (m overlapped, 2H, H-8a, H-9a), 1.24 (m overlapped, 2H, H-7b, H-8b), 1.13 (m, 1H, H-6b), 0.99 (d, *J* = 7.0 Hz, 3H, H_3-16).

^{13}C NMR (175 MHz, $CDCl_3$): 2(*E*) δ_C 184.1 (C-13), 169.4 (C-11), 165.1 (C-15), 162.5 (C-17), 131.4 (C-4), 130.6 (C-3), 109.1 (C-12), 97.3 (C-14), 61.1 (C-20), 52.5 (C-19), 46.4 (C-1), 36.9 (C-5), 35.2 (C-10), 34.4 (C-2), 29.9 (C-6), 28.4 (C-9), 26.1 (C-7), 21.0 (C-8), 20.2 (C-16).

2(*Z*) δ_C 180.7 (C-13), 169.4 (C-11), 166.1 (C-15), 161.1 (C-17), 131.4 (C-4), 130.6 (C-3), 109.1 (C-12), 97.3 (C-14), 61.1 (C-20), 52.3 (C-19), 46.3 (C-1), 36.9 (C-5), 35.2 (C-10), 34.4 (C-2), 29.9 (C-6), 28.4 (C-9), 26.1 (C-7), 21.0 (C-8), 20.2 (C-16).

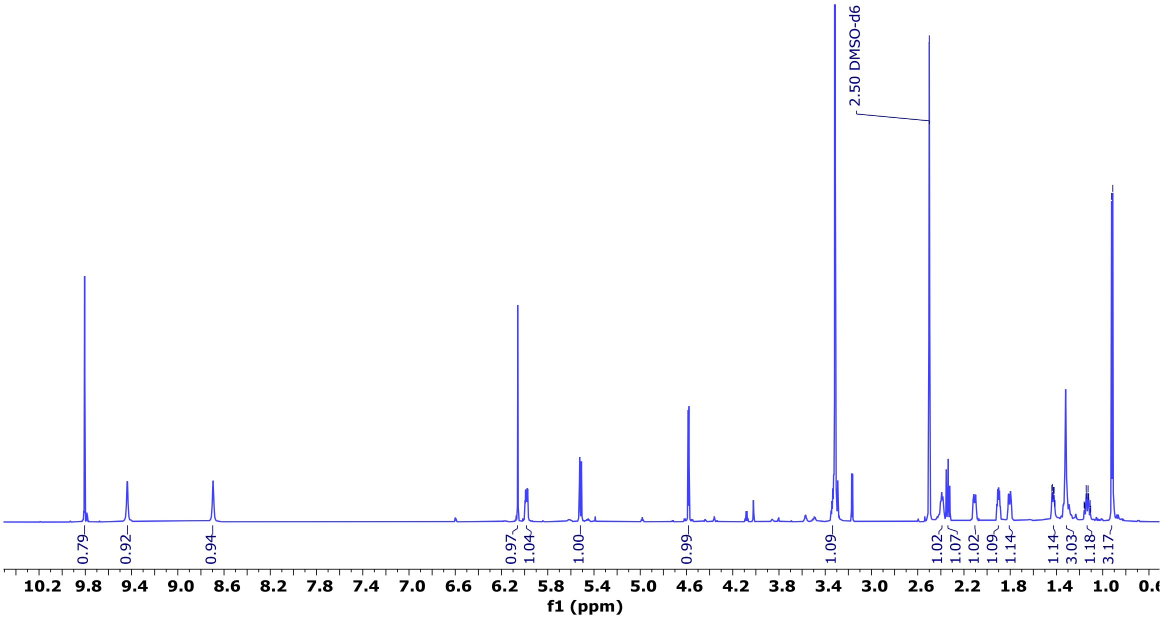

Figure S17. ^{1}H-NMR spectrum of solanapyrone W (**4**) ($CDCl_3$, 700 MHz).

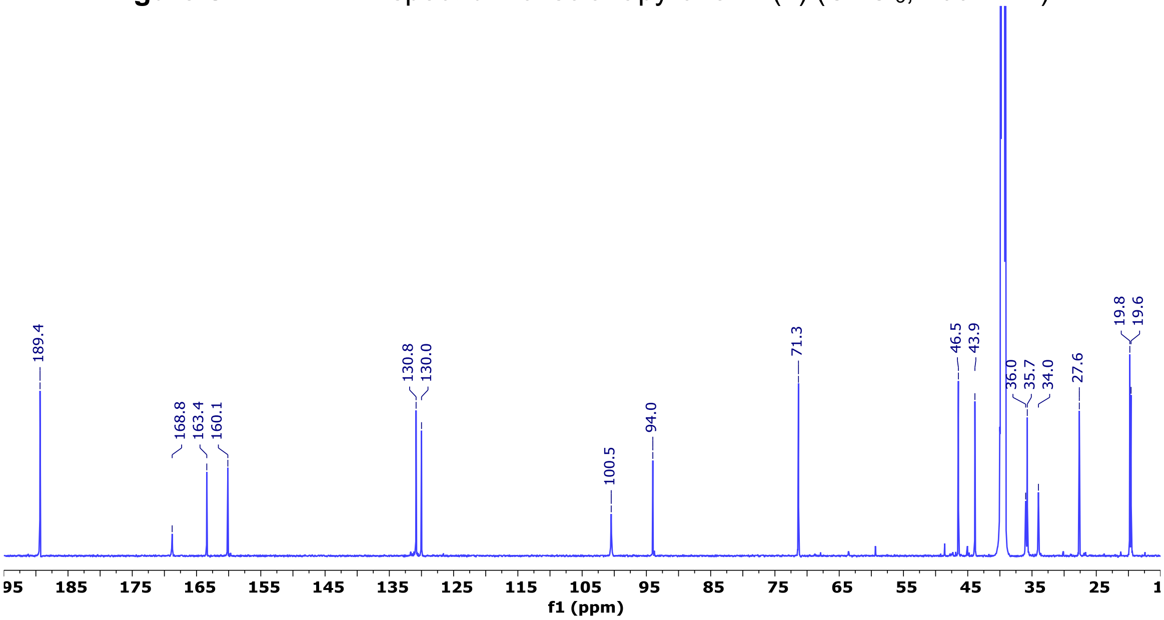

Figure S18. ^{13}C-NMR spectrum of solanapyrone W (**4**) ($CDCl_3$, 175 MHz).

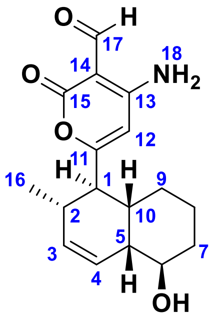

solanapyrone W (**4**): HR-DART^{+} *m/z* 304.1558 $[M+H]^{+}$ calculated for $C_{17}H_{22}NO_4$ 304.1543, Δ +4.8 ppm.

^{1}H NMR (700 MHz, DMSO-d_6): δ_H 9.81 (d, J = 0.6 Hz, 1H, H-17), 9.44 (d, J = 3.7 Hz, 1H, NH-18), 8.69 (d, J = 3.7 Hz, 1H, NH-18), 6.06 (d, J = 0.7 Hz, 1H, H-12), 5.98 (ddd, J = 10.1, 5.0, 2.6 Hz, 1H, H-4), 5.52 (dt, J = 10.1, 1.7 Hz, 1H, H-3), 4.58 (d, J = 5.5 Hz, 1H, 6-OH), 3.33 (overlapped, 1H, H-6), 2.39 (ddt, J = 9.2, 4.4, 2.2 Hz, 1H, H-2), 2.34 (dd, J = 11.7, 10.0 Hz, 1H, H-1), 2.11 (dt, J = 11.6, 3.9 Hz, 1H, H-10), 1.90 (dtt, J = 10.0, 5.0, 1.5 Hz, 1H, H-5), 1.80 (dt, J=13.0, 3.9 Hz, 1H, H-7a), 1.43 (m, 1H, H-8a), 1.32 (m overlapped, 3H, H-8b, H_2-9), 1.13 (qd, J = 12.3, 3.6 Hz, H-7b), 0.92 (d, J = 6.9 Hz, 3H, H_3-16).

^{13}C NMR (DMSO-d_6, 175 MHz): δ_C 189.4 (C-17), 168.8 (C-15), 163.4 (C-11), 160.1 (C-13), 130.8 (C-3), 130.0 (C-4), 100.5 (C-12), 94.0 (C-14), 71.3 (C-6), 46.5 (C-1), 43.9 (C-5), 36.0 (C-10), 35.7 (C-7), 34.0 (C-2), 27.6 (C-8), 19.8 (C-16), 19.6 (C-9).

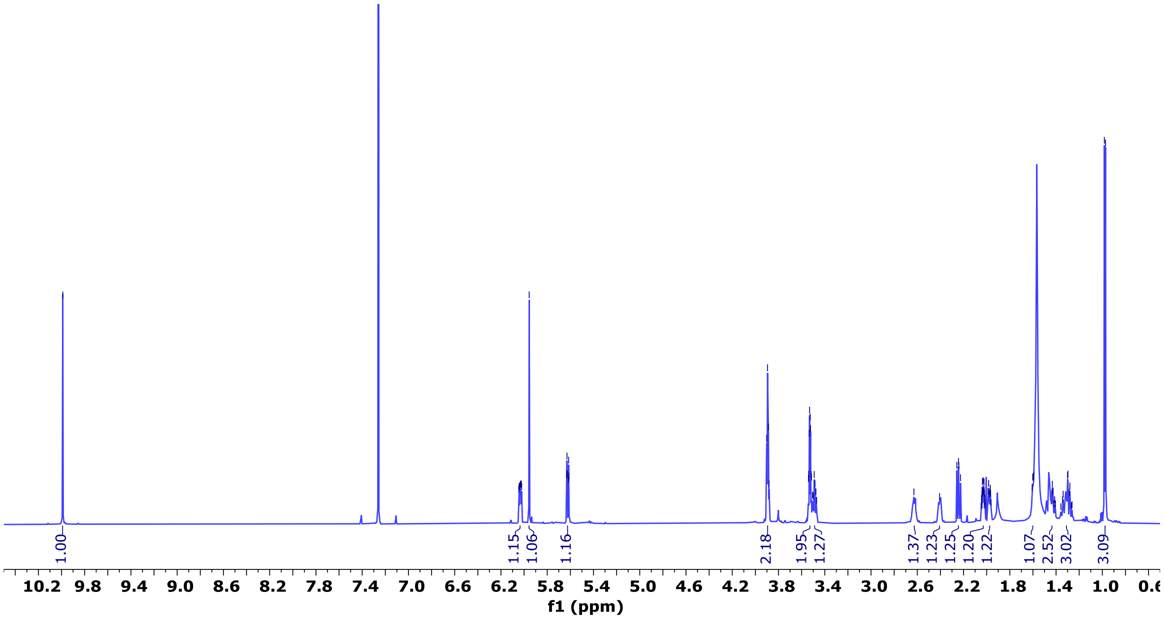

Figure S19. ^{1}H-NMR spectrum of nigrosporapyrone B (**5**) ($CDCl_3$, 700 MHz).

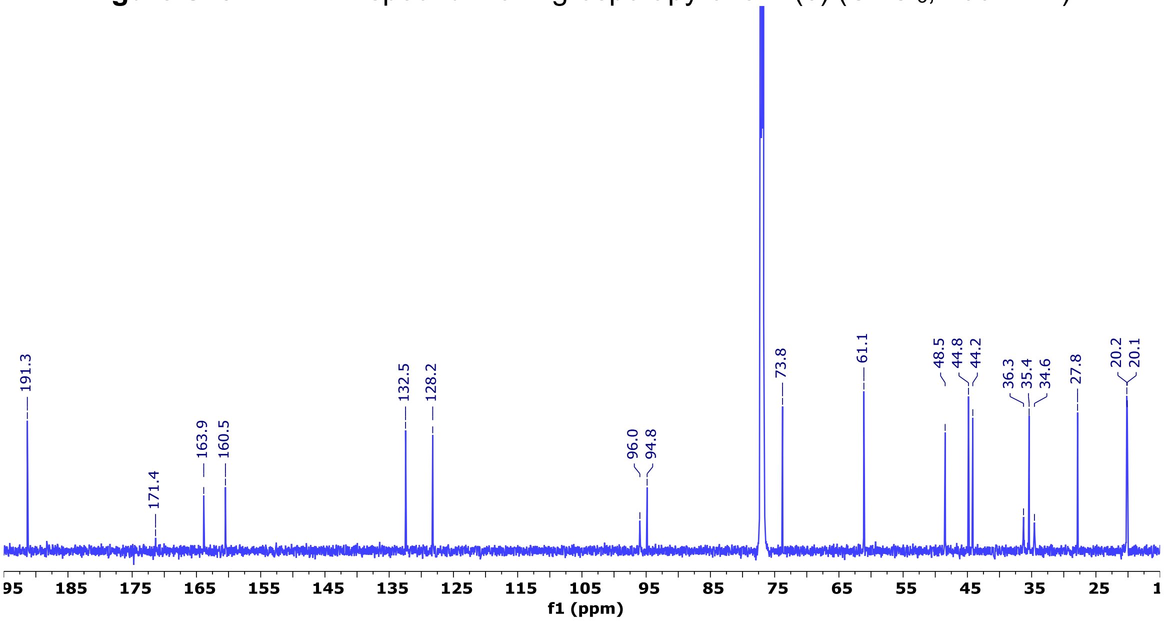

Figure S20. ^{13}C-NMR spectrum of nigrosporapyrone B (**5**) ($CDCl_3$, 175 MHz).

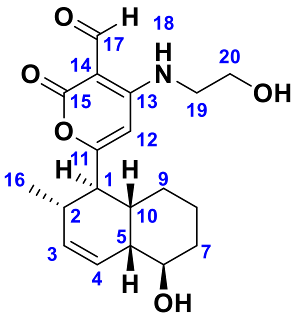

nigrosporapyrone B (**5**): HR-DART^{+} *m/z* 348.1815 $[M+H]^{+}$ calculated for $C_{19}H_{26}NO_5$ 348.1811, Δ +2.7 ppm.

^{1}H NMR (700 MHz, $CDCl_3$): δ_H 10.86 (s-broad, 1H, NH-18), 9.98 (s, 1H, H-17), 6.03 (ddd, *J* = 10.1, 5.1, 2.5 Hz, 1H, H-4), 5.96 (s, 1H, H-12), 5.62 (dd, *J* = 10.1, 1.8 Hz, 1H, H-3), 3.89 (q, *J* = 4.6 Hz, 2H, H_2-20), 3.53 (qd, *J* = 5.2, 1.6 Hz, 2H, H_2-19), 3.49 (td, *J* = 10.5, 4.5 Hz, 1H, H-6), 2.63 (m, 1H, H-2), 2.41 (m, 1H, H-10), 2.24 (dd, *J* = 12.0, 10.0 Hz, 1H, H-1), 2.03 (ddd, *J* = 10.1, 5.1, 1.6 Hz, 1H, H-5), 1.98 (m, 1H, H-7a), 1.60 (m, 1H, H-9a), 1.44 (m, 2H, H_2-8), 1.30 (m overlapped, 2H, H-7b, H-9b), 0.98 (d, *J* = 7.0 Hz, 3H, H_3-16).

^{13}C NMR (175 MHz, $CDCl_3$): δ_C 191.3 (C-17), 171.4 (C-11), 163.9 (C-15), 160.5 (C-13), 132.5 (C-3), 128.2 (C-4), 96.0 (C-12), 94.8 (C-14), 73.8 (C-6), 61.1 (C-20), 48.5 (C-1), 44.8 (C-19), 44.2 (C-5), 36.3 (C-10), 35.4 (C-7), 34.6 (C-2), 27.8 (C-8), 20.2 (C-16), 20.1 (C-9).

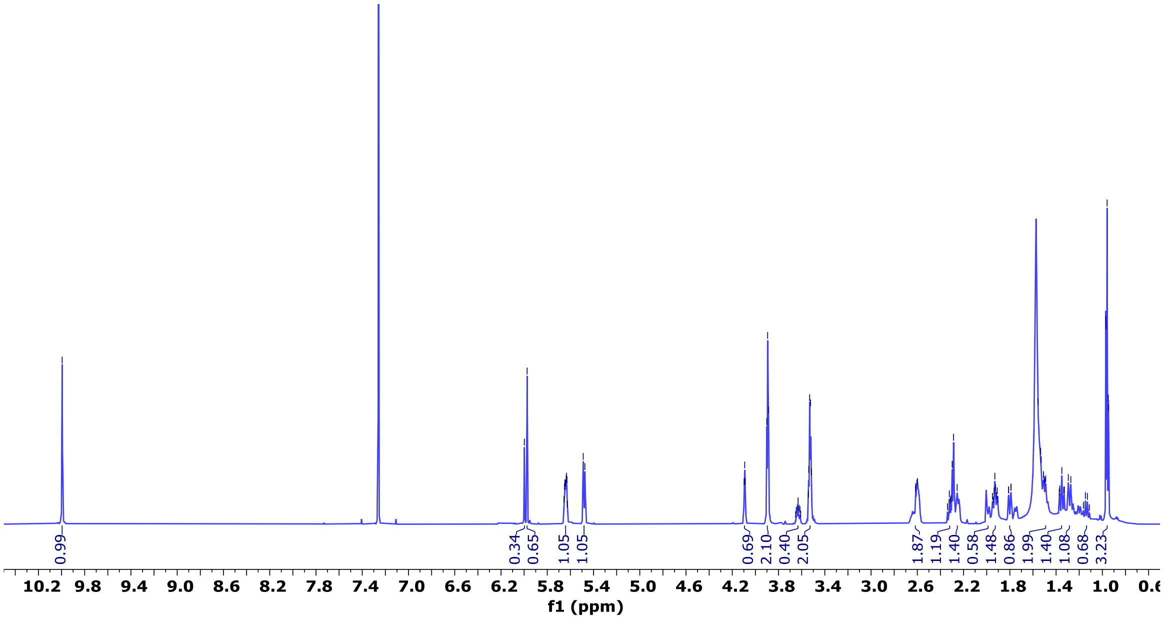

Figure S21. ^{1}H-NMR spectrum of nigrosporapyrone C (**6**) ($CDCl_3$, 700 MHz).

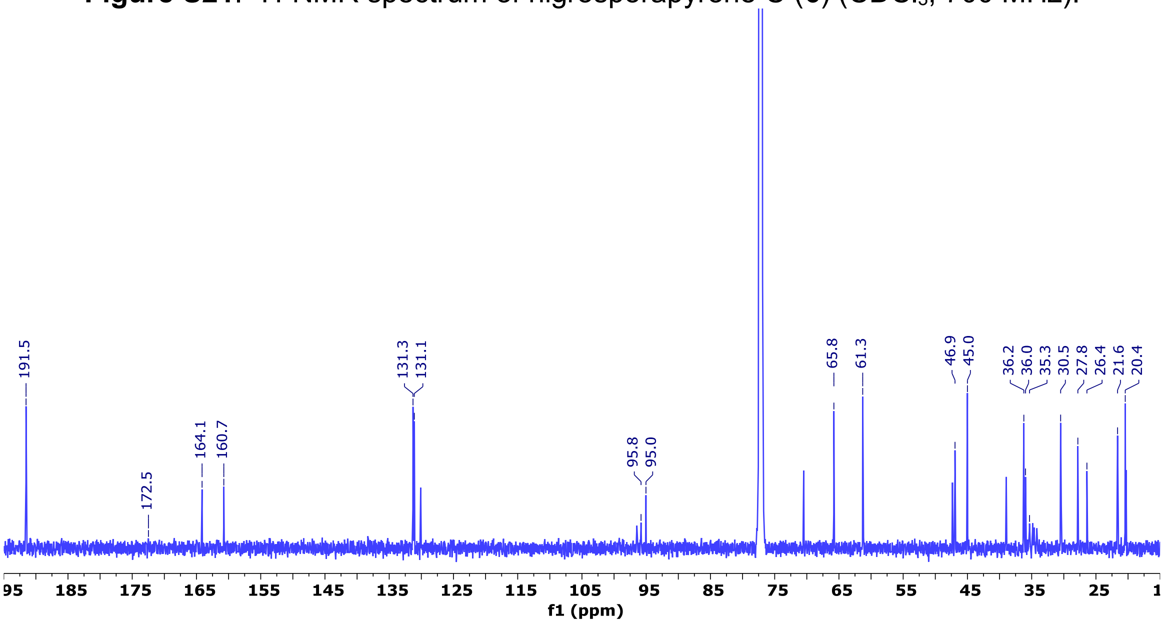

Figure S22. ^{13}C-NMR spectrum of nigrosporapyrone C (**6**) ($CDCl_3$, 175 MHz).

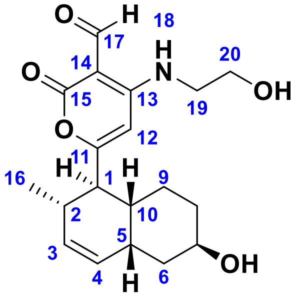

nigrosporapyrone C (**6**): HR-DART$^+$ *m/z* 348.1816 $[M+H]^+$ calculated for $C_{19}H_{26}NO_5$ 348.1805, Δ +3.2 ppm.

^{1}H NMR (700 MHz, $CDCl_3$): δ_H 10.86 (s-broad, 1H, NH-18), 9.99 (s, 1H, H-17), 5.99 (s, 1H), 5.64 (ddd, J = 11.3, 5.7, 3.0 Hz, 1H, H-4), 5.48 (d-broad, J = 10.0 Hz, 1H, H-3), 4.09 (t, J = 3.2 Hz, 1H, H-7), 3.89 (t, J = 5.3 Hz, 2H, H_2-20), 3.53 (q, J = 5.5 Hz, 2H, H_2-19), 2.61 (m overlapped, 2H, H-2, H-5), 2.30 (m, 1H, H-1), 2.25 (m, 1H, H-10), 1.93 (ddt, J = 14.1, 8.4, 4.2 Hz, 1H, H-9a), 1.80 (d, J = 14.2 Hz, 1H, H-6a), 1.50 (m overlapped, 2H, H-8a, H-8b), 1.35 (td, J = 14.0, 2.7 Hz, 1H, H-6b), 1.28 (d-broad, J = 15.2 Hz, 1H, H-9b), 0.96 (t, J = 7.3 Hz, 3H, H_3-16).

^{13}C NMR (175 MHz, $CDCl_3$): δ_C 191.5 (C-17), 172.5 (C-11), 164.1 (C-15), 160.7 (C-13), 131.3 (C-4), 131.1 (C-3), 95.8 (C-12), 95.0 (C-14), 65.8 (C-7), 61.3 (C-20), 46.9 (C-1), 45.0 (C-19), 39.0, 36.2 (C-6), 36.0 (C-10), 35.3 (C-2), 34.8, 34.6, 34.2, 30.5 (C-5), 30.4, 27.8 (C-8), 21.6 (C-9), 20.4 (C-16).

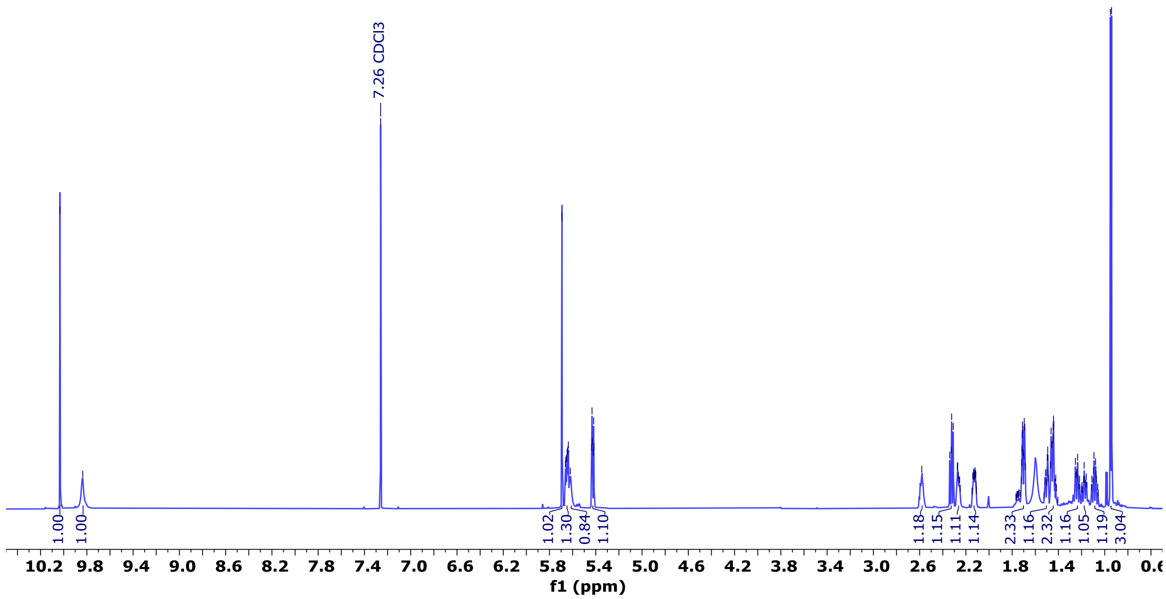

Figure S23. ^{1}H-NMR spectrum of solanapyrone G (**7**) ($CDCl_3$, 500 MHz).

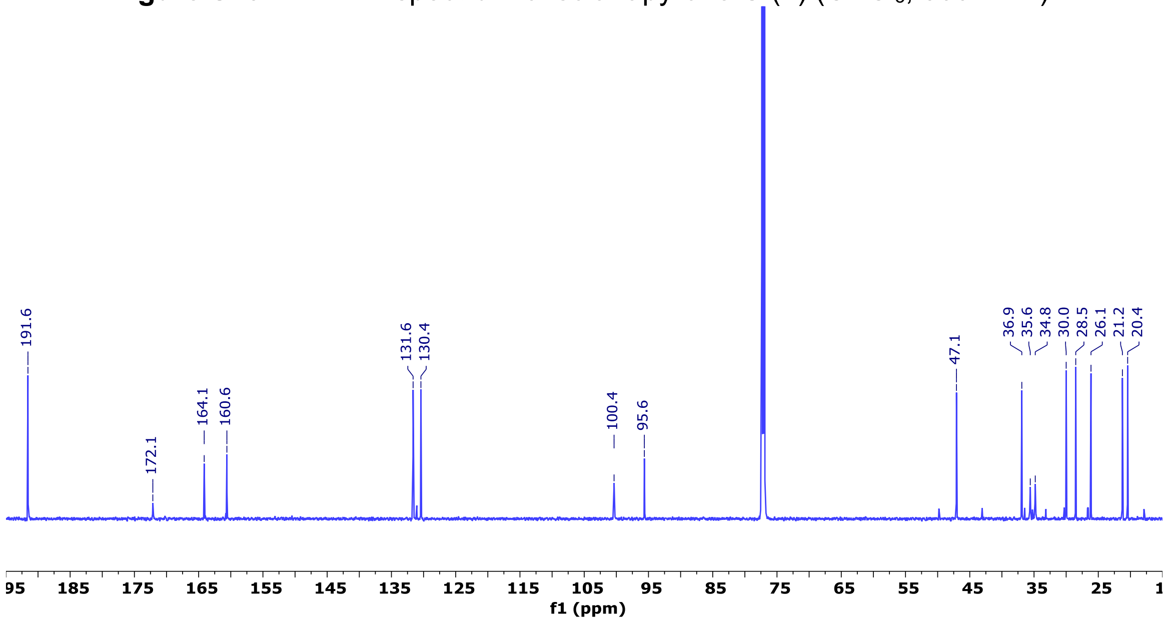

Figure S24. ^{13}C-NMR spectrum of solanapyrone G (**7**) ($CDCl_3$, 125 MHz).

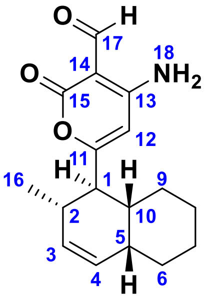

solanapyrone G (**9**): HR-ESI^{+} *m/z* 288.1583 $[M+H]^{+}$ calculated for $C_{17}H_{22}NO_3$ 288.1594, Δ –3.9 ppm.

^{1}H NMR (700 MHz, $CDCl_3$): δ_H 10.03 (s, 1H, H-17), 9.84 (s-broad, 2H, NH-18), 5.69 (d, J = 0.6 Hz, 1H, H-12), 5.65 (ddd, J = 10.1, 5.1, 2.6 Hz, 1H, H-4), 5.43 (dt, J = 9.9, 1.8 Hz, 1H, H-3), 2.58 (m, 1H, H-2), 2.32 (dd, J = 11.8, 9.8 Hz, 1H, H-1), 2.26 (m, 1H, H-10), 2.13 (m, 1H, H-5), 1.72 (m overlapped, 2H, H-6b, H-7b), 1.50 (m, 1H, H-9a), 1.45 (m overlapped, 2H, H-8b, H-9b), 1.23 (ddd, J = 12.5, 9.4, 3.3 Hz, 1H, H-7a), 1.18 (dd, J = 13.3, 3.2 Hz, 1H, H-8a), 1.08 (ddd, J= 12.6, 9.9, 2.9 Hz, 1H, H-6a), 0.95 (d, J = 7.1 Hz, 3H, H_3-16).

^{13}C NMR (175 MHz, $CDCl_3$): δ_C 191.6 (C-17), 172.1 (C-13), 164.1 (C-11), 160.6 (C-15), 131.6 (C-4), 130.4 (C-3), 100.4 (C-12), 95.6 (C-14), 47.1 (C-1), 36.9 (C-5), 35.6 (C-10), 34.8 (C-2), 30.0 (C-6), 28.5 (C-9), 26.1 (C-7), 21.2 (C-8), 20.4 (C-16).

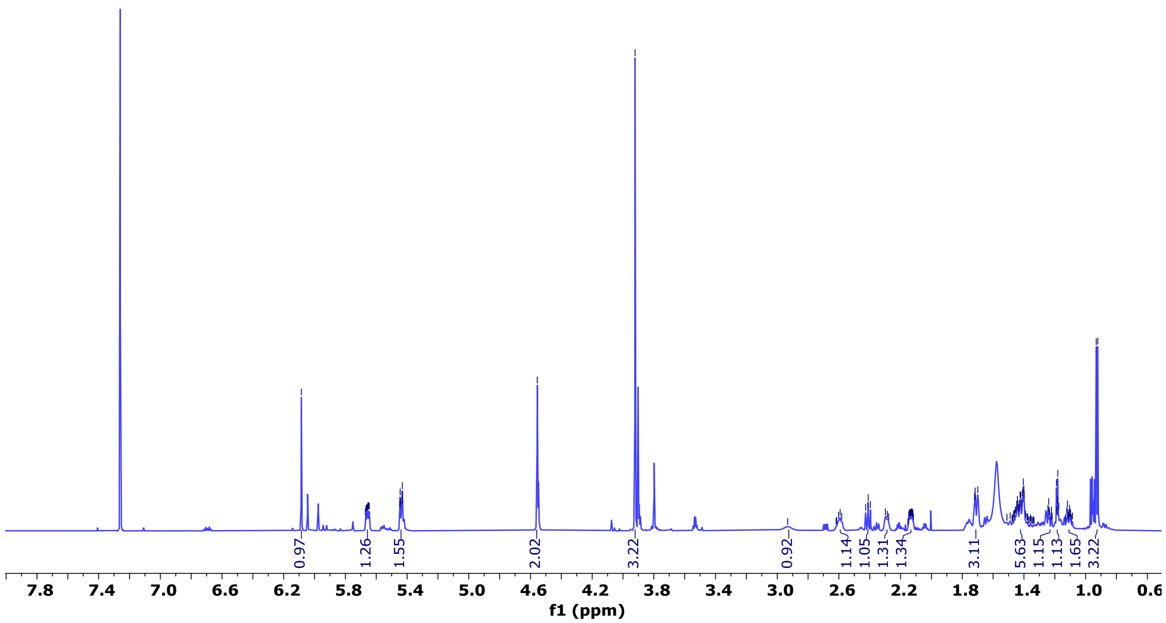

Figure S25. ^{1}H-NMR spectrum of solanapyrone B (**8**) ($CDCl_3$, 700 MHz).

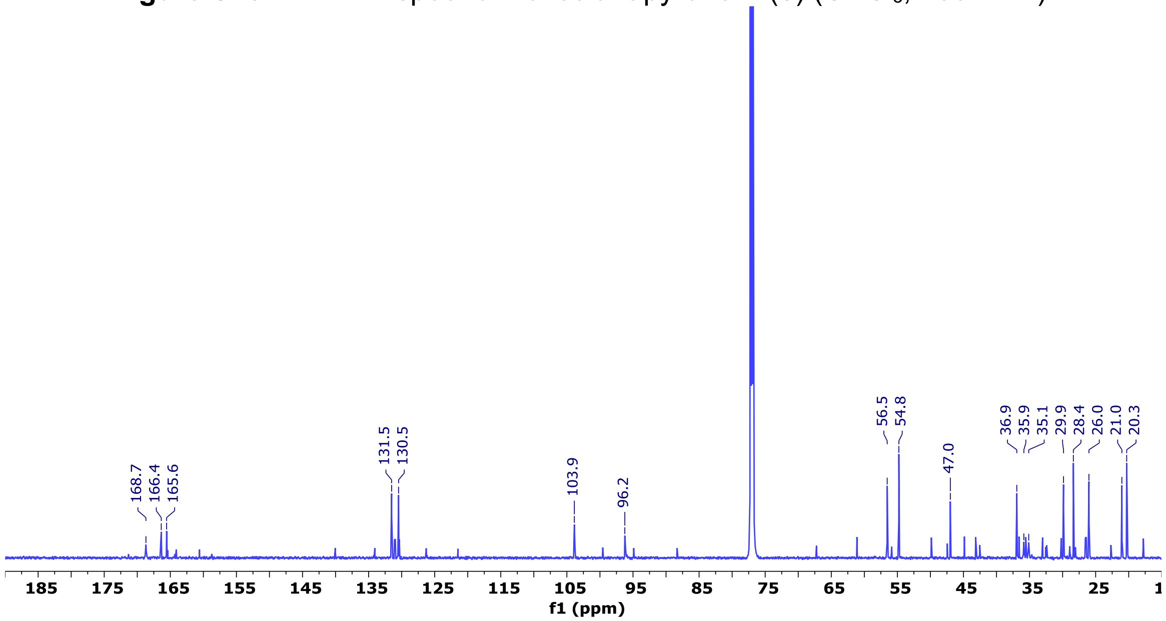

Figure S26. ^{13}C-NMR spectrum of solanapyrone B (**8**) ($CDCl_3$, 175 MHz).

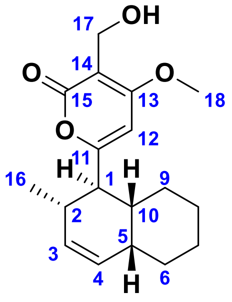

solanapyrone B (**8**): HR-ESI$^+$ *m/z* 305.1735 $[M+H]^+$ calculated for $C_{18}H_{25}O_4$ 305.1747, Δ –4.0 ppm and *m/z* 287.1631 $[M–H_2O]^+$ calculated for $C_{18}H_{23}O_3$ 287.1642, Δ –3.7 ppm.

^{1}H NMR (700 MHz, $CDCl_3$): δ_H 6.09 (s, 1H, H-12), 5.66 (ddd, *J* = 10.0, 5.1, 2.6 Hz, 1H, H-4), 5.44 (dt, *J* = 9.9, 1.8 Hz, 1H, H-3), 4.52 (s, 1H, H-17), 3.92 (s, 3H, H_3-18), 2.59 (m, 1H, H-2), 2.41 (dd, *J* = 11.8, 10.1 Hz, 1H, H-1), 2.29 (m, 1H, H-5), 2.13 (m, 1H, H-10), 1.71 (m overlapped, 2H, H-6b, H-7b), 1.42 (m overlapped, 3H, H-8b, H_2-9), 1.23 (dt, *J* = 12.1, 3.2 Hz, 1H, H-7a), 1.18 (dd, *J* = 6.2, 4.5 Hz, 1H, H-8a), 1.10 (m, 1H, H-6a), 0.93 (d, *J* = 7.0 Hz, 3H, H_3-16).

^{13}C NMR (175 MHz, $CDCl_3$): δ_C 168.7 (C-11), 166.4 (C-13), 165.6 (C-15), 131.5 (C-4), 130.5 (C-3), 103.9 (C-14), 96.2 (C-12), 56.5 (C-18), 54.8 (C-17), 47.0 (C-1), 36.9 (C-10), 35.9 (C-5), 35.1 (C-2), 29.9 (C-6), 28.4 (C-9), 26.0 (C-7), 21.0 (C-8), 20.3 (C-16).

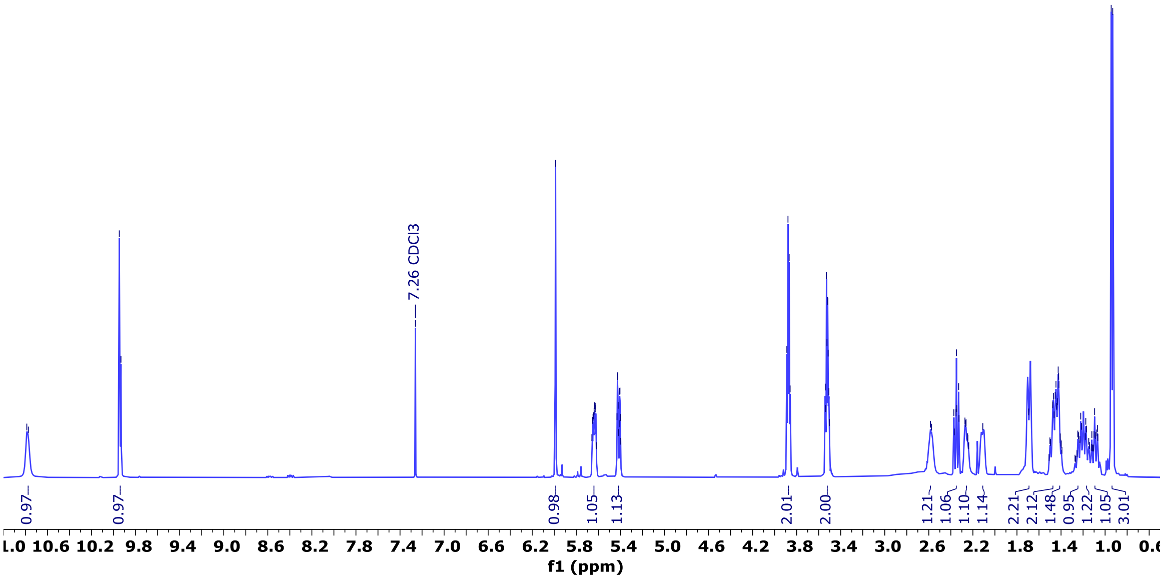

Figure S27. ^{1}H-NMR spectrum of solanapyrone C (**9**) ($CDCl_3$, 500 MHz).

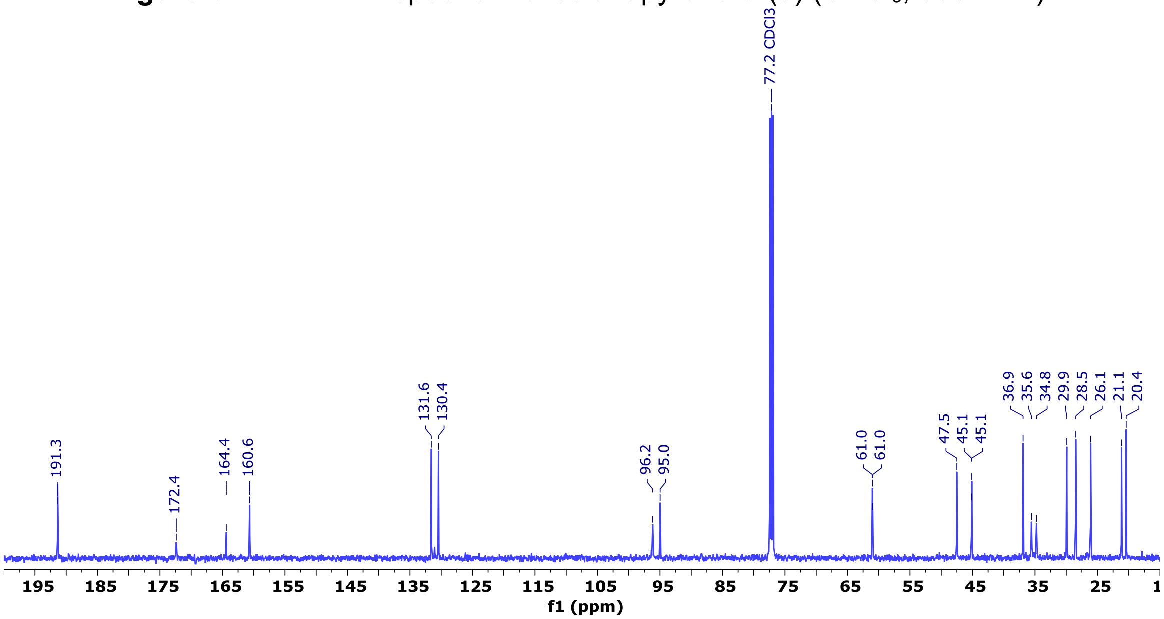

Figure S28. ^{13}C-NMR spectrum of solanapyrone C (**9**) ($CDCl_3$, 125 MHz).

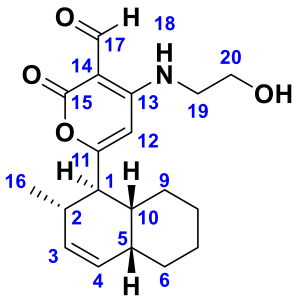

solanapyrone C (**9**): HR-ESI$^+$ *m/z* 332.1842 $[M+H]^+$ calculated for $C_{19}H_{26}NO_4$ 332.1856, Δ –4.3 ppm.

^{1}H NMR (500 MHz, $CDCl_3$): δ_H 10.78 (s-broad, 1H, NH-18), 9.94 (s, 1H, H-17), 5.99 (s, 1H, H-12), 5.64 (ddt, *J* = 10.5, 5.5, 2.8 Hz, 1H, H-4), 5.41 (ddt, *J* = 9.9, 3.4, 1.8 Hz, 1H, H-3), 3.87 (q, *J* = 4.8 Hz, 2H, H_2-20), 3.52 (qd, *J* = 5.4, 2.8 Hz, 2H, H_2-19), 2.58 (m, 1H, H-2), 2.35 (ddd, *J* = 11.7, 9.8, 1.8 Hz, 1H, H-1), 2.26 (m, 1H, H-10), 2.11 (m, 1H, H-5), 1.69 (m overlapped, 2H, H-6b, H-7b), 1.47 (td, *J* = 15.0, 2.8 Hz, 2H, H-9a, H-9b), 1.42 (m, 1H, H-8b), 1.24 (dt, *J* = 12.9, 3.3 Hz, 1H, H-7a), 1.16 (dd, *J* = 13.5, 2.8 Hz, 1H, H-8b), 1.10 (tt, *J* = 11.8, 2.4 Hz, 1H, H-6a), 0.94 (d, *J* = 7.0 Hz, 3H, H_3-16).

^{13}C NMR (125 MHz, $CDCl_3$): δ_C 191.3 (C-17), 172.4 (C-13), 164.4 (C-11), 160.6 (C-15), 131.6 (C-4), 130.4 (C-5), 96.2 (C-12), 95.0 (C-14), 61.0 (C-20), 47.5 (C-1), 45.1 (C-19), 36.9 (C-5), 35.6 (C-10), 34.8 (C-2), 29.9 (C-6), 28.5 (C-9), 26.1 (C-7), 21.1 (C-8), 20.4 (C-16).

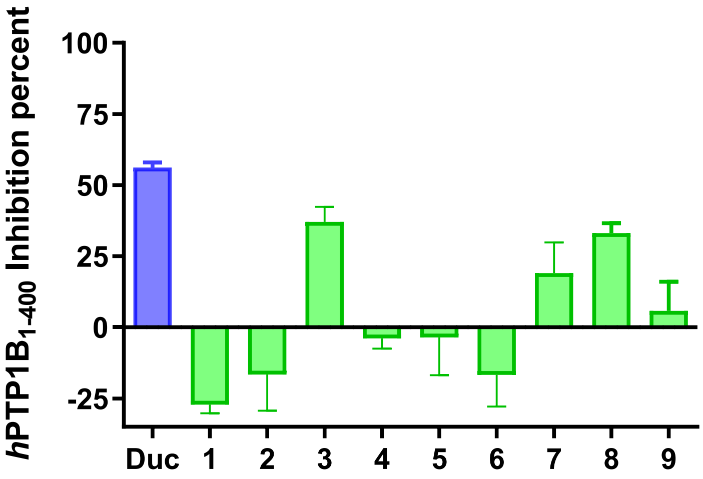

Figure S29. *h*PTP1B$_{1\text{-}400}$ inhibition percentage for compounds **1**–**9**. Duclauxin (Duc) was employed as a positive control at 20 μM, compounds were tested at 100 μM.

Table S1. Inhibitory activity against *Acinetobacter baumannii* strain A564 by some compounds of *Nigrospora* sp. IQ-064.

Compound	Growth inhibition (%)
Solanapyrone G (**7**)	15.98 ± 5.12
Solanapyrone C (**9**)	31.61 ± 1.71
Positive control<br>Colistin in water at 20 ppm	99.95 ± 0.22
Positive control<br>Gentamicin in water at 64 ppm	29.80 ± 2.79
Negative control	0

Compounds were evaluated at a concentration of 100 ppm in dimethyl sulfoxide. The average and standard deviation of at least 3 replicates are reported.

Table S2. ADME properties predicted for compounds **1-9**.

Compound	1	2	3	4	5	6	7	8	9
**Physicochemical Properties**									
Formula	$C_{19}H_{27}NO_6$	$C_{19}H_{25}NO_5$	$C_{19}H_{25}NO_4$	$C_{17}H_{21}NO_4$	$C_{19}H_{25}NO_5$	$C_{19}H_{25}NO_5$	$C_{17}H_{21}NO_3$	$C_{18}H_{24}O_4$	$C_{19}H_{25}NO_4$
MW	365.42	347.41	331.41	303.35	347.41	347.41	287.35	304.38	331.41
#Heavy atoms	26	25	24	22	25	25	21	22	24
#Aromatic heavy atoms	6	6	0	6	6	6	6	6	6
Fraction Csp3	0.68	0.68	0.58	0.53	0.58	0.58	0.53	0.61	0.58
#Rotatable bonds	5	5	4	2	5	5	2	3	5
#H-bond acceptors	6	5	4	4	5	5	3	4	4
#H-bond donors	4	2	2	2	3	3	1	1	2
MR	96.77	93.42	91.13	84.27	95.14	95.14	83.1	85.93	93.97
TPSA	120	92.07	75.63	93.53	99.77	99.77	73.3	59.67	79.54
**Lipophilicity**									
iLOGP	1.86	2.29	3.03	1.5	2.15	1.93	1.89	3.09	2.33
XLOGP3	1.69	2.68	3.6	2.05	2.03	1.85	3.58	3.68	3.56
WLOGP	0.93	1.97	2.09	2.11	1.73	1.73	3.14	3.08	2.76
MLOGP	0.23	1.03	1.57	1.29	0.95	0.95	2.12	2.44	1.76
Silicos-IT Log P	1.57	2.81	2.2	1.83	2.06	2.06	2.74	3.19	2.95
Consensus Log P	1.26	2.16	2.5	1.76	1.78	1.7	2.69	3.1	2.67
**Water Solubility**									
ESOL Log S	-3.01	-3.53	-3.9	-3.08	-3.12	-3.01	-3.96	-4.05	-3.99
ESOL Solubility (mg/ml)	3.56e-01	1.03e-01	4.18e-02	2.51e-01	2.63e-01	3.42e-01	3.18e-02	2.72e-02	3.37e-02
ESOL Solubility (mol/l)	9.75e-04	2.95e-04	1.26e-04	8.28e-04	7.58e-04	9.84e-04	1.11e-04	8.93e-05	1.02e-04
ESOL Class	Soluble	Soluble	Soluble	Soluble	Soluble	Soluble	Soluble	Moderately soluble	Soluble
Ali Log S	-3.82	-4.27	-4.88	-3.64	-3.75	-3.57	-4.81	-4.62	-4.92
Ali Solubility (mg/ml)	5.47e-02	1.88e-02	4.42e-03	6.91e-02	6.14e-02	9.43e-02	4.50e-03	7.25e-03	4.02e-03
Ali Solubility (mol/l)	1.50e-04	5.42e-05	1.33e-05	2.28e-04	1.77e-04	2.72e-04	1.57e-05	2.38e-05	1.21e-05
Ali Class	Soluble	Moderately soluble	Moderately soluble	Soluble	Soluble	Soluble	Moderately soluble	Moderately soluble	Moderately soluble
Silicos-IT LogSw	-2.75	-3.65	-2.6	-2.48	-3.1	-3.1	-3.29	-3.65	-3.92
Silicos-IT Solubility (mg/ml)	6.48e-01	7.85e-02	8.34e-01	1.00e+00	2.76e-01	2.76e-01	1.46e-01	6.77e-02	4.02e-02

**Compound**	**1**	**2**	**3**	**4**	**5**	**6**	**7**	**8**	**9**
**Water Solubility**									
Silicos-IT Solubility (mol/l)	1.77e-03	2.26e-04	2.52e-03	3.30e-03	7.94e-04	7.94e-04	5.09e-04	2.22e-04	1.21e-04
Silicos-IT class	Soluble	Soluble	Soluble	Soluble	Soluble	Soluble	Soluble	Soluble	Soluble
**Pharmacokinetics**									
GI absorption	High	High	High	High	High	High	High	High	High
BBB permeant	No	No	Yes	No	No	No	Yes	Yes	No
Pgp substrate	Yes	Yes	No	Yes	Yes	Yes	No	No	Yes
CYP1A2 inhibitor	No	No	No	No	No	No	No	No	No
CYP2C19 inhibitor	No	No	No	No	No	No	No	No	No
CYP2C9 inhibitor	No	No	Yes	No	No	No	No	No	No
CYP2D6 inhibitor	No	Yes	No	No	No	No	No	Yes	No
CYP3A4 inhibitor	No	No	No	No	No	No	No	No	Yes
log Kp (cm/s)	-7.33	-6.52	-5.77	-6.69	-6.98	-7.11	-5.51	-5.54	-5.79
**Druglikeness**									
Lipinski #violations	0	0	0	0	0	0	0	0	0
Ghose #violations	0	0	0	0	0	0	0	0	0
Veber #violations	0	0	0	0	0	0	0	0	0
Egan #violations	0	0	0	0	0	0	0	0	0
Muegge #violations	0	0	0	0	0	0	0	0	0
Bioavailability Score	0.55	0.55	0.56	0.55	0.55	0.55	0.55	0.55	0.55
**Medicinal Chemistry**									
PAINS #alerts	0	0	0	0	0	0	0	0	0
Brenk #alerts	1	2	3	2	2	2	2	1	2
Leadlikeness #violations	1	0	1	0	0	0	1	1	1
Synthetic Accessibility	5.09	4.96	5.21	4.75	4.99	4.96	4.63	4.79	4.83

Table S3. PASS analysis for compounds **1–9**. The predicted probabilities of activity (Pa) and inactivity (Pi) are shown for each compound. Only activities with Pa values ≥ 0,700 were considered for this analysis.

**Compound**	CDP-glycerol glycerophosphotransferase inhibitor		Phosphatase inhibitor		CYP3A1 substrate		CYP2C11 substrate		Caspase 3 stimulant		Antiparasitic		Respiratory analeptic		Antieczematic	
	Pa	Pi	Pa	Pi	Pa	Pi	Pa	Pi	Pa	Pi	Pa	Pi	Pa	Pi	Pa	Pi
**1**	0,779	0,035	0,703	0,012												
**2**	0,739	0,007	0,719	0,049												
**3**																
**4**	0,800	0,030	0,728	0,008	0,701	0,009	0,701	0,007	0,769	0,007			0,736	0,012		
**5**	0,763	0,038	0,706	0,012												
**6**	0,763	0,038	0,700	0,013	0,717	0,023					0,708	0,005				
**7**	0,771	0,036	0,713	0,010			0,737	0,006								
**8**					0,703	0,009			0,744	0,009					0,703	0,045
**9**	0,729	0,046									0,725	0,005				
